# Integrin CD11b negatively regulates Mincle-induced signaling via the Lyn–SIRPα–SHP1 complex

**DOI:** 10.1038/emm.2017.256

**Published:** 2018-02-05

**Authors:** Quanri Zhang, Wook-Bin Lee, Ji-Seon Kang, Lark Kyun Kim, Young-Joon Kim

**Affiliations:** 1 Department of Integrated Omics for Biomedical Science, Graduate School, Yonsei University, Seoul, Republic of Korea; 2 Department of Biochemistry, College of Life Science and Biotechnology, Yonsei University, Seoul, Republic of Korea; 3 Severance Biomedical Science Institute and BK21 PLUS Project to Medical Sciences, Seoul, Republic of Korea; 4 Severance Institute for Vascular and Metabolic Research, Gangnam Severance Hospital, Yonsei University College of Medicine, Seoul, Republic of Korea

## Abstract

During mycobacteria infection, anti-inflammatory responses allow the host to avoid tissue damage caused by overactivation of the immune system; however, little is known about the negative modulators that specifically control mycobacteria-induced immune responses. Here we demonstrate that integrin CD11b is a critical negative regulator of mycobacteria cord factor-induced macrophage-inducible C-type lectin (Mincle) signaling. CD11b deficiency resulted in hyperinflammation following mycobacterial infection. Activation of Mincle by mycobacterial components turns on not only the Syk signaling pathway but also CD11b signaling and induces formation of a Mincle–CD11b signaling complex. The activated CD11b recruits Lyn, SIRPα and SHP1, which dephosphorylate Syk to inhibit Mincle-mediated inflammation. Furthermore, the Lyn activator MLR1023 effectively suppressed Mincle signaling, indicating the possibility of Lyn-mediated control of inflammatory responses. These results describe a new role for CD11b in fine-tuning the immune response against mycobacterium infection.

## Introduction

The hallmark of *Mycobacterium tuberculosis* (Mtb) infection is the formation of a granuloma, a compact aggregate of immune cells.^1^ The granuloma has been thought to function as a host defense mechanism to prevent further spread of Mtb; however, recent studies suggest that the granuloma can also shelter the bacteria and ensure persistence of these organisms in a latent form.^2, 3^ Therefore, clearance of the mycobacteria that persist in the granuloma is required for efficient clearance of the infection.

Granuloma formation is initiated by an orchestrated production of cytokines and chemokines coupled with the upregulation of selectins and integrins on immune cells to recruit and activate different populations of leukocytes.^4^ As granuloma formation progresses, the intense proinflammatory responses are suppressed by negative modulators, to prevent excessive granuloma formation.^5^ The most prominent anti-inflammatory cytokine involved in the downregulation of granuloma formation is interleukin (IL)-10, which antagonizes the activity of IL-17 and interferon (IFN)-γ, thereby lessening the protective immune responses of macrophages.^6, 7^ Additionally, IL-10 may inhibit antigen presentation by dendritic cells (DCs) via blockade of major histocompatibility complex molecules.^8^ This compromised immune environment may, therefore, enable the bacteria to evade host immune surveillance and survive for a long time in the lungs, ultimately leading to a chronic infection. Hence, understanding the protective mechanism of negative regulators of granuloma formation will elucidate key targets for the development of immune therapies to fight Mtb infection.

Mtb carries diverse pathogen-associated molecular patterns (PAMPs) that can initiate an inflammatory response in the host. Among these PAMPs, the virulent cord factor trehalose-6,6-dimycolate (TDM) is specifically recognized by macrophage-inducible C-type lectin (Mincle), and this cord factor alone leads to a granulomatous response through the robust production of nitric oxide (NO) and various proinflammatory cytokines and chemokines, including IL-6, tumor necrosis factor (TNF)-α and monocyte chemoattractant protein-1.^9, 10, 11^ Additionally, TDM stimulation critically enhances the cellular adhesion of neutrophils by increasing their surface expression of integrin CD11b/CD18.^12^ Amplified integrin surface expression allows neutrophils to infiltrate into and accumulate around infected sites, enabling recruitment of additional neutrophils to kill the bacteria. Thus Mincle appears to have a key role in the fight of leukocytes against mycobacteria infection. Although the activation of the proinflammatory response by Mincle has been studied extensively, the negative mediators that specifically restrain Mincle signaling during granuloma formation remain to be elucidated.

The integrin heterodimer CD11b/CD18 mainly functions in cell adhesion and migration during inflammation.^13^ Intriguingly, recent studies in CD11b-deficient mice suggest a broad crosstalk between CD11b and various pattern recognition receptor-mediated pathways. Specifically, CD11b activated by Toll-like receptor 4 (TLR4) signaling targets myeloid differentiation primary response gene 88 (Myd88) and TIR domain-containing adapter-inducing interferon-β (TRIF) for proteasome degradation, thereby negatively regulating the TLR4 signal in peripheral macrophages.^14^ Moreover, CD11b can interfere with the function of T and B cells by suppressing the differentiation of T_H_17 cells and inhibiting B-cell receptor signaling.^15, 16^ Considering that CD11b is upregulated in TDM-activated neutrophils and has a role in the negative regulation of TLR signaling, the relatively static function of macrophages in the granuloma may result from the negative regulation of Mincle by CD11b during granuloma formation.

In the present study, we demonstrate that TDM-challenged CD11b-deficient mice developed more severe granulomas with increased recruitment of leukocytes and increased production of proinflammatory cytokines. In macrophages, the absence of CD11b led to increased cytokine production and release of NO and reactive oxygen species (ROS) upon TDM stimulation, as well as to enhanced cytokine production against *Mycobacterium bovis* Bacillus Calmette-Guérin (BCG) infection. With respect to neutrophils, CD11b was indispensable for adhesion, and CD11b deficiency resulted in increased cytokine levels. We found that activated CD11b formed a signaling complex with activated Mincle that recruited Lyn kinase, SIRPα and SHP1; this complex regulated the phosphorylation of the Mincle downstream target Syk, thereby suppressing Mincle-dependent inflammatory responses. Therefore, CD11b functions as a negative modulator of TDM–Mincle signaling by dephosphorylating Syk kinase via the Lyn–SIRPα–SHP1 complex.

## Material and methods

### Animals

C57BL/6 mice, aged 6–7 weeks, were purchased from Orient Bio. CD11b^−/−^ mice were obtained from Jackson Laboratories (Bar Harbor, ME), and Mincle^−/−^ mice (Clec4eMNA) were obtained from the Consortium for Functional Glycomics. The animals were backcrossed for nine generations onto the C57BL/6 background. All mice were maintained in a specific pathogen-free facility at the Laboratory Animal Research Center at Yonsei University, Seoul, Republic of Korea. Protocols were approved by the Institutional Animal Care and Use Committees of the Laboratory Animal Research Center at Yonsei University (permit number: IACUC-A-201410-317-01).

### Cell

Bone marrow-derived macrophages (BMMs) were prepared by culturing BM cells with 20% (v/v) L929 culture supernatant in basic Dulbecco’s modified Eagle medium (ThermoFisher Scientific, Grand Island, NY, USA) supplemented with 20% fetal bovine serum (Gibco, ThermoFisher Scientific, Grand Island, NY, USA), 50 U ml^−1^ penicillin and 50 mg ml^−1^ streptomycin for 7 days. BMDCs were differentiated by culturing BM cells with 15 ng ml^−1^ recombinant granulocyte-macrophage colony-stimulating factor in Dulbecco’s modified Eagle medium for 10 days. BM neutrophils were purified directly from BM cells by 53/63/76% three-layer Percoll gradient centrifugation as described^12^ and cultured in RPMI basic medium supplemented with 10% fetal bovine serum and penicillin/streptomycin. The immortalized BMM (iBMM) cell line was obtained from BEI Resources, NIAID NIH (NR-9456) (no. NR-9456).

For TDM stimulation, 50 μg ml^−1^ TDM (Sigma-Aldrich, St Louis, MO, USA) was added to culture plates before cell plating. Cells were primed with 10 ng ml^−1^ IFN-γ (IFN-γ (Pierce, Rockford, IL, USA), 100 ng ml^−1^ Pam3CSK4 (Invivogen, San Diego, CA, USA) and 10 ng ml^−1^ ultrapure lipopolysaccharide (LPS; InvivoGen) for the indicated times. For the inhibitor assay, the indicated inhibitors were added 30 min before stimulation. PP1 (Src family kinase inhibitor, 529579, 5 μM), PP2 (Src family kinase inhibitor, 529573, 100 nM), Syk Inhibitor (574711, 10 μM) and SB203580 (559389, 10 μM) were purchased from Calbiochem (La Jolla, CA, USA). AG490 (T3434, 25 μM), SP600125 (S5567, 10 μM) and BHA (B1253, 50 μM) were obtained from Sigma. Parthenolide (0610, 5 μM), U0126 (1144, 10 μM), wortmannin (1232, 10 μM), Lyn peptide inhibitor (2265, 2 μM) and Lyn activator MLR1023 (4582, 1 ng ml^−1^) were purchased from Tocris (Ellisville, MO, USA). Fibrinogen from Sigma (F3879, 15 μg ml^−1^) was diluted in phosphate-buffered saline (PBS) and coated on plates at 4 °C overnight. Then the fibrinogen was aspirated and the plate was washed three times with PBS before cell plating.

### Enzyme-linked immunosorbent assay, immunoblot analysis and immunoprecipitation

IL-6 and TNF-α secretion in culture supernatants was measured using an enzyme-linked immunosorbent assay (ELISA) kit (BioLegend, San Diego, CA, USA). For immunoblot analysis, cells were lysed for 15 min at 4 °C in RIPA lysis buffer (100 mM Tris-HCl, pH 8.0, 50 mM NaCl, 5 mM EDTA, 0.5% NP-40, 1% Triton X-100, 50 mM β-glycerophosphate, 50 mM NaF, 0.1 mM Na_3_VO_4_ and 0.5% sodium deoxycholate) with protease inhibitor cocktail (Roche, Indianapolis, IN, USA). Following lysis, suspensions were centrifuged at 1300 *g* for 15 min at 4 °C to remove nuclei. Then the proteins were separated by sodium dodecyl sulfate-polyacrylamide gel electrophoresis and transferred to nitrocellulose membranes. The membranes were developed with Amersham enhanced chemiluminescence reagents, followed by detection of the signal using the ImageQuant LAS4000 system GE Healthcare (Piscataway, NJ, USA). For immunoprecipitation assays, cells were stimulated with TDM for the indicated times. Then the cells were washed and resuspended in NP-40 lysis buffer (50 mM Tris-HCl, pH 7.5, 1 mM EGTA, 1 mM EDTA, pH 8.0, 50 mM NaF, 1 mM sodium glycerophosphate, 5 mM pyrophosphate, 0.27 M sucrose, 0.5% NP-40, 0.1 mM phenylmethylsulfonyl fluoride, 0.1% 2-mercaptoethanol, 1 mM Na_3_VO_4_ and protease inhibitor cocktail (Roche)). After removing nuclei by centrifugation at 1300 *g* for 15 min at 4 °C, the cell extracts were incubated with V5 agarose or agarose-A beads with Lyn or SHP1 antibodies overnight at 4 °C. After pull-down, the agarose was washed three times in ice-cold lysis buffer and proteins were eluted by boiling in sodium dodecyl sulfate sample buffer. The precipitated proteins were subjected to immunoblot analysis as described above. The antibodies used in this study are listed in Supplementary Table 1.

### Adhesion assay

Neutrophils (3 × 10^5^ cells per well) were labeled by incubation with 5 μg ml^−1^ calcein- acetoxymethyl ester at 37 °C for 30 min, washed with PBS and resuspended in RPMI medium containing 10% fetal bovine serum. Cells were then plated in 48-well tissue culture plates for TDM stimulation. After 6 h of adhesion, non-adherent cells were washed away with PBS aspiration, and adherent cells were imaged under a fluorescent microscope. The percentage of adherent cells was determined by comparison of fluorescence measured by a microplate reader Tecan Infinite Pro 200. (TECAN, Switzerland) at 492 nm before and after washing.

### ROS assay

ROS production was measured with carboxy-H_2_DCFDA dye (Molecular Probes, Eugene, OR, USA). Macrophages (3 × 10^5^ cells per well) and neutrophils (1 × 10^5^ cells per well) were seeded in 96-well plates and preincubated with 10 μM H_2_DCFDA in PBS for 30 min. After washing in PBS, cells were stimulated with TDM. After 6 and 18 h of treatment, oxidized DCFDA was analyzed at 495 nm/520 nm using a Multilabel Plate Reader (Victor5, Perkin Elmer, Waltham, MA, USA). The data were normalized to the negative control, which consisted of unchallenged cells.

### NO measurement

NO concentration was measured by classic colorimetric Griess reaction.^17^ Culture supernatants were incubated with an equal volume of Griess reagent (Sigma-Aldrich, G4410) for 5 min at RT. The absorbance was determined at 570 nm with a Perkin Elmer 550S spectrophotometer. Prepared sodium nitric oxide (1–100 μM) was used to generate a standard curve for calculating sample NO concentrations.

### Plasmid construction and site-directed mutagenesis

The open reading frame of complementary DNA (cDNA) for CD11b, CD18, Mincle, FcRγ, Syk, Lyn, Shp1, Shp2 and Ship-1 were obtained from BMM by PCR amplification using the primers listed in Supplementary Table 2. Then the expression units were digested with the indicated enzymes and inserted into the enzyme-digested vectors.

The SHP1 D419A and C453S dominant-negative mutants were constructed using a Site-Directed Mutagenesis Kit (Stratagene, Agilent Technologies, La Jolla, CA, USA) according to the manufacturer’s instructions. Primers used are listed in Supplementary Table 2.

### TDM-induced pulmonary granulomas

To elicit pulmonary granuloma formation, 9–11-week-old mice were injected intravenously through the tail vein with 100 μl of a water-in-oil emulsion containing 100 μg of TDM (Sigma). On day 7 postchallenge, mice were killed, lungs were weighed and the lung weight index was calculated as described previously.^18, 19^ A portion of the fresh lung was subjected to flow cytometry to assess the leukocyte infiltration just after killing. The large lobe of the right lung was fixed in 10% formaldehyde for hematoxylin and eosin staining. Other lung sections were homogenized and frozen for future analysis by ELISA or quantitative reverse transcription-coupled PCR (qRT-PCR).

### Air pouch model

Briefly, air pouches were established on the dorsal sides of 9–10-week-old wild-type (WT) and CD11b^−/−^ mice by subcutaneous injection of 3 ml of sterile air on day 0. A second injection of 1.5 ml of sterile air into the pouch was performed on day 3. On day 7, 200 μl of a water-in-oil emulsion containing 50 μg of TDM was injected into the pouch cavity, while an emulsion without TDM was injected into the control animals. Approximately 24 h after the final injection, mice were killed, and the pouch cavities were washed with PBS. The wash fluid was harvested for leukocyte population analysis by flow cytometry and cytokine measurement by ELISA.

### Flow cytometric analysis

A portion of the lungs was weighed, incubated in 2 mg ml^−1^ collagenase D (Roche) and 40 U ml^−1^ DNase I (Roche) and dispersed by passage through 70 mm mesh. After red blood cell lysis, viable cells were counted and incubated with fluorescence-conjugated antibodies for labeling. The following specific FACS antibodies (BD Pharmingen, San Diego, CA, USA) were used: Gr-1 (RB6-8C5), CD11b (M1/70), Ly6G (1A8), CD3e (145-2C11) and CD19 (1D3). To determine CD11b and CD18 expression on neutrophils, purified neutrophils were incubated with anti-mouse CD11b (M1/70) and CD18 (C71/16) (BD Pharmingen). After incubation, cells were analyzed on a FACS Calibur instrument (BD Biosciences). The isotype control antibodies used in this experiment were obtained from BD Pharmingen.

### *In situ* PLA

Protein–protein interactions were investigated using the Duolink *In Situ* Red Starter Kit (Sigma, DUO92105) according to the manufacturer’s instructions. Immortalized BMMs were transfected using Lipofectamine for 24 h, and 1 × 10^3^ cells in 200 μl of medium were seeded onto 8-well chamber slides coated with TDM. After 24 h, cells were fixed in 4% paraformaldehyde for 15 min. Fluorescence was detected with a LSM 700 (Carl Zeiss, Oberkochen, Germany) confocal microscope, and signal intensities were quantified with Photoshop CS (Photoshop, Adobe, Mountain View, CA, USA). Antibodies used in the PLA assay are listed in Supplementary Table 1. And we provide the expression levels of transfected proteins in Supplementary Figure 13.

### CRISPR-Cas9-dependent gene knockout in iBMM cells

Guide RNAs were designed at the site http://crispr.mit.edu. Then synthesized single-strand guide RNAs (listed in Supplementary Table 2) were annealed to form gRNA oligo duplexes and ligated into digested lentiCRISPR v2 vectors (Addgene, Cambridge, MA, USA plasmid #52961; a gift from Feng Zhang) according to the Addgene’s instruction. Lentivirus was generated by co-transfecting HEK 293FT cells with lentiCRISPR v2, psPAX2 (packaging) and pMD2.G (envelope). After 48 h, virus-containing supernatants were collected and added onto the iBMM cells along with polybrene (8 μg ml^−1^, Sigma). After another 24 h for virus transduction, cells were selected with puromycin (2 μg ml^−1^), and single-cell colonies were further selected by plating in 96-well plates. Gene knockout colonies were validated by immunoblot. Colonies that still expressed the target proteins were used as negative control lines.

### BCG infection

Macrophages were seeded in 48-well plates (2.0 × 10^5^ cells per well) and incubated overnight. Cells were then infected with BCG at a multiplicity of infection of 10 for 4 h. Non-internalized bacteria were washed away with PBS, and cells were incubated with fresh Dulbecco’s modified Eagle medium. At 24, 48 and 72 h after infection, media was collected for the analysis of cytokine levels by ELISA.

### Quantitative reverse transcription-coupled PCR

Total RNA from cells and tissues was extracted with TRIzol reagent (ThermoFisher Scientific) according to the manufacturer’s instructions. Then cDNA was synthesized using SuperScript II reverse transcriptase (ThermoFisher Scientific) with oligo(dT) primers. Expression of individual genes was determined by real-time PCR using a Bio-Rad CFX (Bio-Rad, Hercules, CA, USA) and quantified by normalizing to the housekeeping gene *Gapdh* by the change-in-cycling-threshold (ΔΔC_T_) method. Primers used are listed in Supplementary Table 2.

### Statistical analysis

Software Prism 6.0 (GraphPad Software Inc. La Jolla, CA, USA) was used to run unpaired two-tailed *t*-tests with a 95% confidence interval for the calculation of *P-*values.

## Results

### Increased cytokine production against mycobacteria in CD11b-deficient mice

**Figure 1:**
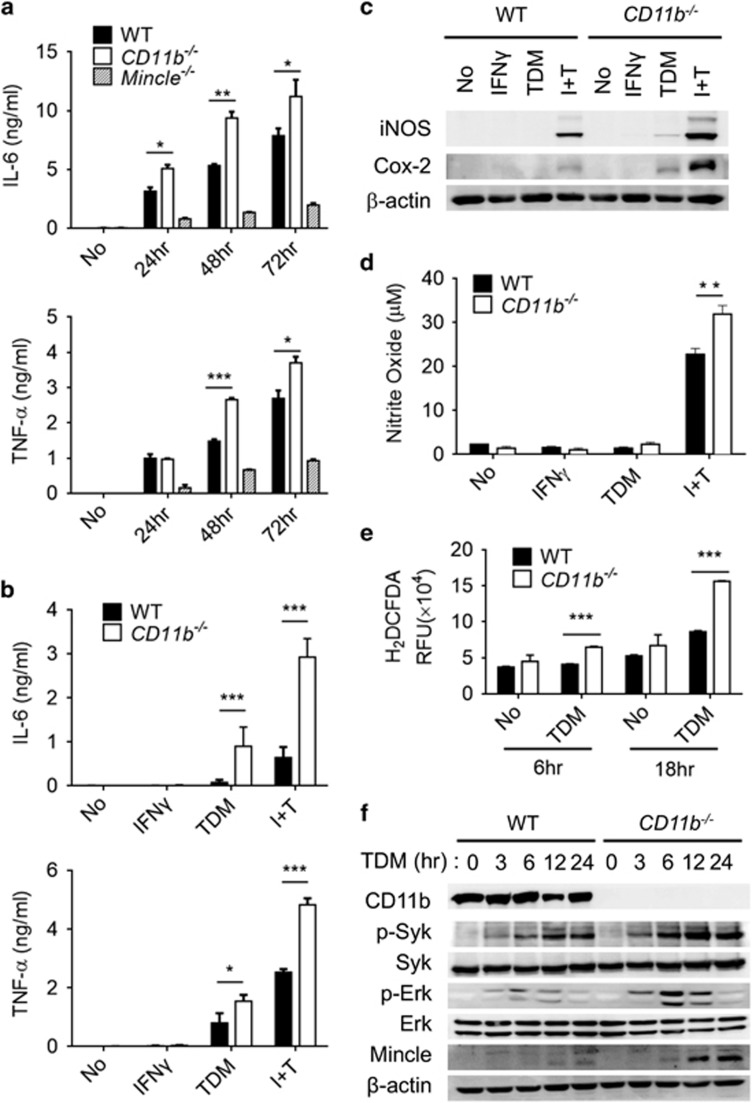
CD11b deficiency enhances the macrophage response to BCG infection and TDM stimulation. (**a**) BMMs from WT, CD11b^−/−^ and Mincle^−/−^ mice were infected with BCG at an MOI of 5, and cell culture supernatants were collected at the indicated time points. Secreted cytokines (IL-6 and TNF-α) were assayed by ELISA. (**b**–**d**) WT and CD11b^−/−^ BMMs were stimulated with 10 ng ml^−1^ IFN-γ or 50 μg ml^−1^ TDM or co-stimulated with TDM and IFN-γ (I+T), for 24 h. (**b**) IL-6 and TNF-α cytokine levels were determined by ELISA. (**c**) Expression of iNOS and Cox-2 was determined by immunoblot. (**d**) NO generation was measured with Griess reagent. (**e**) Induction of ROS was determined by H_2_DCFDA assay 6 and 18 h after stimulation with 50 μg ml^−1^ TDM. (**f**) Phosphorylation of Syk and Erk in WT and CD11b^−/−^ BMMs was analyzed by immunoblot at the indicated times. The β-actin protein level was used as the loading control in immunoblot assays. Data are representative of at least three independent experiments. *P<0.05, **P<0.01, ***P<0.0001 (two-tailed unpaired Student’s *t*-test).

To examine the involvement of the integrin receptor in anti-mycobacterial infection, BMMs from WT, CD11b-deficient (CD11b^−/−^) and Mincle-deficient (Mincle^−/−^) mice were challenged with BCG. BCG infection of WT BMMs induced the secretion of high levels of inflammatory cytokines such as IL-6 and TNF-α (Figure 1a); however, Mincle^−/−^ BMMs were defective in the secretion of these cytokines in response to BCG treatment, indicating that Mincle is the major pattern recognition receptor that mediates cytokine release during BCG infection. By contrast, CD11b deficiency caused hyperinduction of inflammatory cytokines in response to BCG treatment, indicating that CD11b exerts an inhibitory effect on anti-mycobacterial immune signaling. Because CD11b is known to mediate inflammation by regulating leukocyte adhesion,^20^ we asked whether the defective cellular adhesion in CD11b-deficient BMMs is responsible for the abnormal production of cytokines. To this end, we compared the phagocytosis efficiencies of WT and mutant BMMs using fluorescent beads. Interestingly, all BMMs tested exhibited comparable phagocytic ability, even in the presence of TDM-coated particles (Supplementary Figure 1). Together, these results indicate that CD11b deficiency may affect anti-mycobacterial cytokine production, which is not related to phagocytosis.

### Enhanced TDM–Mincle signaling in CD11b-deficient macrophages

Because TLR signaling is independent of CD11b in BMMs,^21^ the hyperinduction of inflammatory cytokines in CD11b-deficient BMMs upon BCG infection appeared to mainly be dependent on Mincle, which recognizes the major cell wall component of BCG. Thus, we investigated the potential role of CD11b in the direct regulation of Mincle using a Mincle-specific ligand, TDM. TDM stimulation caused greater production of IL-6 and TNF-α in CD11b^−/−^ BMMs than in WT BMMs (Figure 1b). The disparity between the WT and the CD11b^−/−^ phenotype was further increased when the macrophages were primed with IFN-γ. Therefore, the hyperinflammatory cytokine production in response to BCG in the CD11b-deficient BMMs appeared to have resulted from abnormal regulation of the Mincle signaling pathway.

To confirm the effect of CD11b deletion on Mincle signaling, we examined the expression of key molecules that are specifically regulated by Mincle, such as inducible nitric oxide synthase and Cox-2.^22^ Indeed, inducible nitric oxide synthase and Cox-2 were highly expressed in the TDM-stimulated CD11b^−/−^ macrophages, while expression of these factors was not detectable in the TDM-stimulated WT BMMs. These discrepancies were further increased under IFN-γ-primed conditions that induced high Mincle expression (Figure 1c). Consistently, NO and NADPH oxidase-dependent ROS were produced at higher levels in CD11b^−/−^ macrophages than in WT macrophages upon TDM stimulation (Figures 1d and e). Measurement of mRNA by qRT-PCR revealed that diverse inflammatory cytokines (*Il6*, *Tnf*, *Il1a* and *Il1b*), chemokines (*Ccl2*, *Cxcl2* and *Cxcl10*) and signaling molecules (*Nos2* and *Mmp3*), as well as anti-inflammatory cytokines (*Il10* and *Ifnb*), were highly upregulated in the CD11b^−/−^ macrophages (Supplementary Figure 2).

To further examine the involvement of CD11b in Mincle signaling, the activation of molecules downstream of Mincle was monitored. TDM stimulation induced extracellular signal-regulated kinase (Erk)1/2 and spleen tyrosine kinase (Syk) phosphorylation specifically, and the activation of these molecules was further enhanced in CD11b^−/−^ BMMs compared with WT BMMs (Figure 1f). To rule out the possibility of Mincle-dependent kinase activation via positive feedback due to higher Mincle expression in CD11b^−/−^ macrophages, WT and CD11b^−/−^ macrophages were primed with TLR agonists to induce similar levels of Mincle expression, as described previously.^23^ Both LPS and Pam3, a TLR2 agonist, induced a similar level of Mincle protein expression, and both stimuli failed to induce Syk or Erk1/2 phosphorylation in the absence of TDM treatment. TDM treatment of the primed BMMs, however, activated Syk and Erk1/2 phosphorylation robustly, with significantly stronger activation in the CD11b^−/−^ BMMs than in the WT cells (Supplementary Figure 3). Together, these lines of evidence indicate that CD11b regulates the Mincle pathway specifically during mycobacterial infection.

### Hyperinflammatory immune response of CD11b^−/−^ mice following TDM stimulation

**Figure 2:**
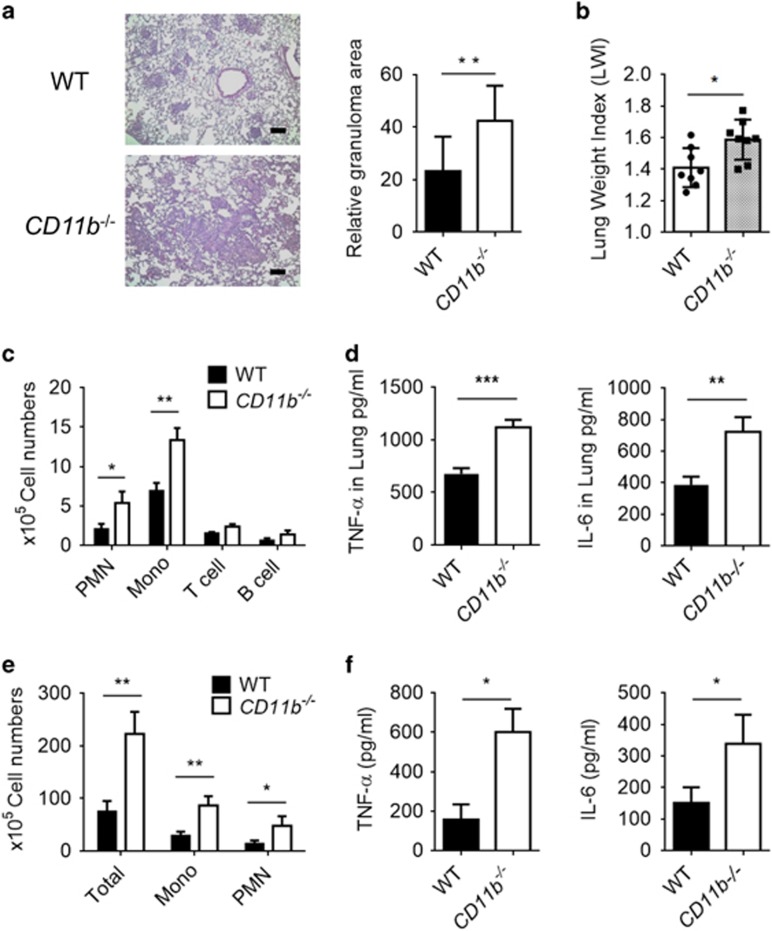
Absence of CD11b leads to more severe granuloma formation and hyper-recruitment of inflammatory cells *in vivo.* (**a**–**d**) WT and CD11b^−/−^ mice (*n*=8) were intravenously injected with 3.75 mg kg^−1^ TDM in an oil-in-water emulsion and killed at 7 days post-TDM challenge. (**a**) Lungs were isolated and weighed to calculate the lung weight index (LWI). (**b**) Lungs were stained with hematoxylin and eosin (H&E) for histology analysis. Scale bars, 100 mm. (**c**) Flow cytometry was performed for leukocyte subset analysis with distinct markers for monocytes and macrophages (Mo/Ma; CD11b+ Ly6G−), neutrophils (PMN; CD11b+ Ly6G+), T cells (CD3+) and B cells (CD19+). (**d**) The lung homogenates were analyzed by ELISA for IL-6 and TNF-α production. (**e**, **f**) For the air pouch model, mice (*n*=8) were dorsolaterally injected with sterile air on days 0 and 3 followed by injection of 2.5 mg kg^−1^ of a TDM emulsion on day 7. On day 8, wash fluid from the pouches was assessed by flow cytometry for leukocyte subsets, as described above (**e**), and the IL-6 and TNF-α cytokine levels were determined by ELISA (**f**). *P<0.05, **P<0.01, ***P<0.0001 (two-tailed unpaired Student’s *t*-test). Data are representative of two independent experiments. (**b**–**f**, mean and s.d. of eight mice per group.)

To examine the inhibitory effect of CD11b on Mincle signaling under physiological conditions, WT and CD11b^−/−^ mice were challenged with TDM to induce a lung granuloma that mimics mycobacterial infection. Intravenous injection of TDM induced granuloma formation in both WT and CD11b^−/−^ mice, but TDM induced the formation of more severe granulomas in the lungs of the *CD11b*-deficient mice than in the lungs of WT mice. The relative granuloma area and the lung weight index, which indicate the severity of the inflammation, were higher in the lungs of CD11b^−/−^ mice than in the lungs of the WT control group (Figures 2a and b). The numbers of recruited neutrophils and monocytes were significantly elevated in the lungs of CD11b^−/−^ mice, in concert with a slight increase in T and B cells compared with the WT mice (Figure 2c). Reflecting the hyperinflammatory conditions in the TDM-stimulated CD11b^−/−^ mice, higher RNA and protein levels of proinflammatory cytokines such as TNF-α and IL-6 were detected in lung homogenates from CD11b^−/−^ mice (Figure 2d and Supplementary Figure 4). In addition, qRT-PCR analysis revealed a similar upregulation of inflammatory cytokines (*Il1a* and *Il12a*), chemokines (*Ccl2*, *Cxcl2* and *Cxcl10*) and signaling molecules (*Nos2* and *Mmp3*) in the lungs of CD11b-deficient mice (Supplementary Figure 4).

To confirm the effect of CD11b on TDM-induced granulomatous tissue formation, the inflammatory activity of TDM was assessed in WT and CD11b^−/−^ mice using an air pouch model.^24^ Similar to the results obtained with intravenous injection of TDM, we observed significantly increased levels of leukocytes and inflammatory cytokines (TNF-α and IL-6) in the TDM-stimulated air pouches in CD11b^−/−^ mice compared with that in WT mice (Figures 2e and f). Taken together, these results indicate that TDM challenge leads to exaggerated inflammatory responses in CD11b^−/−^ mice, suggesting that CD11b is required for the downregulation of inflammation and granuloma formation induced by Mincle activation.

### Impaired adhesion but increased TDM signaling in CD11b^−/−^ neutrophils

**Figure 3:**
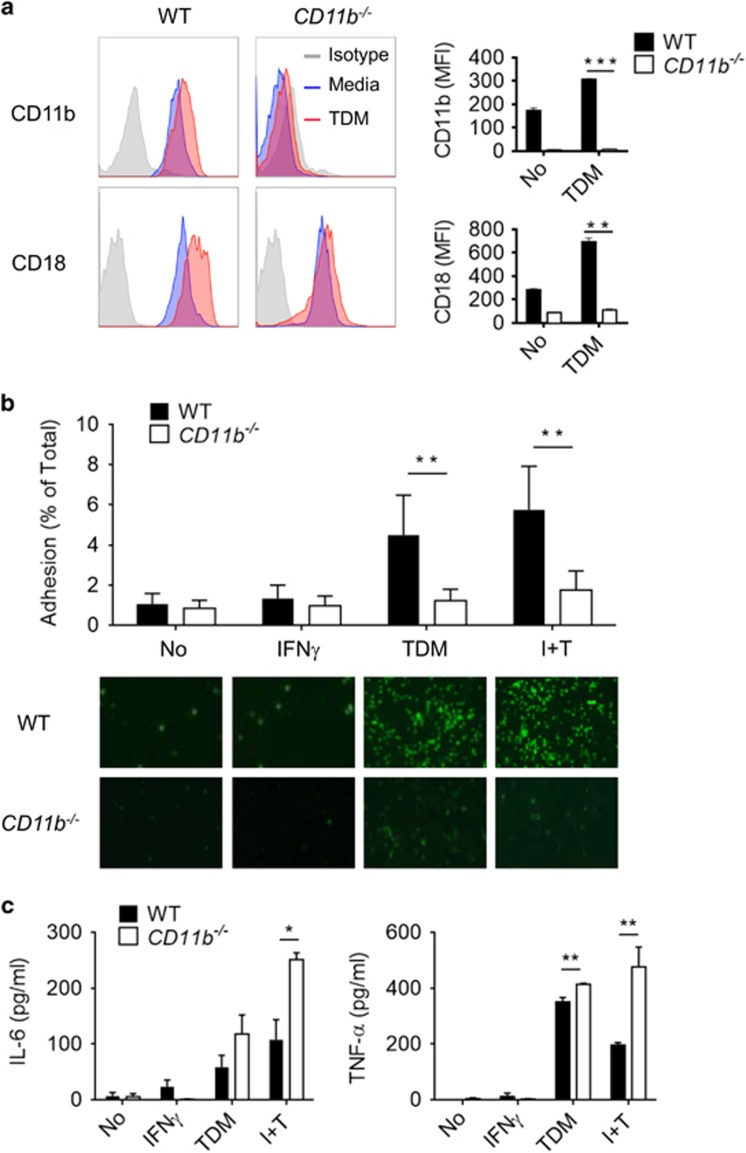
CD11b-deficient neutrophils exhibit impaired adhesion but increased activity upon Mincle activation. (**a**) Bone marrow (BM) neutrophils from WT and CD11b-deficient mice were treated with 50 μg ml^−1^ TDM, and the surface expression of CD11b and CD18 was determined by flow cytometry 24 h after stimulation. (**b**) WT and CD11b^−/−^ BM neutrophils were prelabeled with calcein acetoxymethyl ester 6 h before treatment with 50 μg ml^−1^ TDM treatment, with or without priming with 10 ng ml^−1^ IFN-γ (I+T). Adhered cells were imaged with a fluorescent microscope (bottom panel, × 40). The percentage of adherent cells was determined by measuring the fluorescence with a microplate reader at 492 nm before and after washing (**b**, top panel). (**c**) IL-6 and TNF-α levels were assayed 24 h after TDM stimulation. Data are representative of three independent experiments. *P<0.05, **P<0.01, ***P<0.0001 (two-tailed unpaired Student’s *t*-test).

To determine whether CD11b can inhibit Mincle signaling in other cell types, the anti-TDM response was examined in neutrophils derived from WT and CD11b^−/−^ mice. As described previously,^12^ TDM stimulation increased the surface expression of CD11b and its binding partner CD18 in the WT BM-derived neutrophils. On the other hand, CD11b-deficient BM neutrophils exhibited much less CD18 surface expression than WT BM neutrophils, and this expression did not increase following TDM treatment (Figure 3a). In accordance with the increased CD11b/CD18 surface expression, WT BM-derived neutrophils showed increased adhesion to a TDM-coated surface, while no apparent cell adhesion was observed from the CD11b^−/−^ BM-derived neutrophils (Figure 3b). Despite the reduced levels of adhesion, the secretion of TNF-α and IL-6 was higher in CD11b^−/−^ BM neutrophils than in WT BM neutrophils, and cytokine production was further enhanced in the CD11b^−/−^ neutrophils by IFN-γ treatment (Figure 3c). To determine whether the altered cytokine production may have resulted from an effect of CD11b deficiency on neutrophil survival, apoptosis induced by TDM treatment was evaluated in WT and CD11b^−/−^ neutrophils. TDM stimulation increased neutrophil apoptosis in WT and CD11b^−/−^ BM neutrophils at a similar rate (Supplementary Figure 5a). Taken together, these findings demonstrate that CD11b is required for cell adhesion and inhibition of cytokine production upon TDM stimulation but does not impact TDM-induced apoptosis in neutrophils.

Although CD11b has been shown to be required for the induction of MyD88-dependent TLR signaling in DCs,^21^ WT and CD11b^−/−^ DCs stimulated with a synthetic analog of TDM, trehalose-6,6-dibehenate, showed no apparent difference in the secretion of the inflammatory cytokines TNF-α and IL-6 (Supplementary Figure 5b). Therefore, CD11b appears to have an inhibitory role in Mincle signaling in macrophages and neutrophils specifically.

### CD11b interacts with Mincle specifically upon TDM treatment

**Figure 4:**
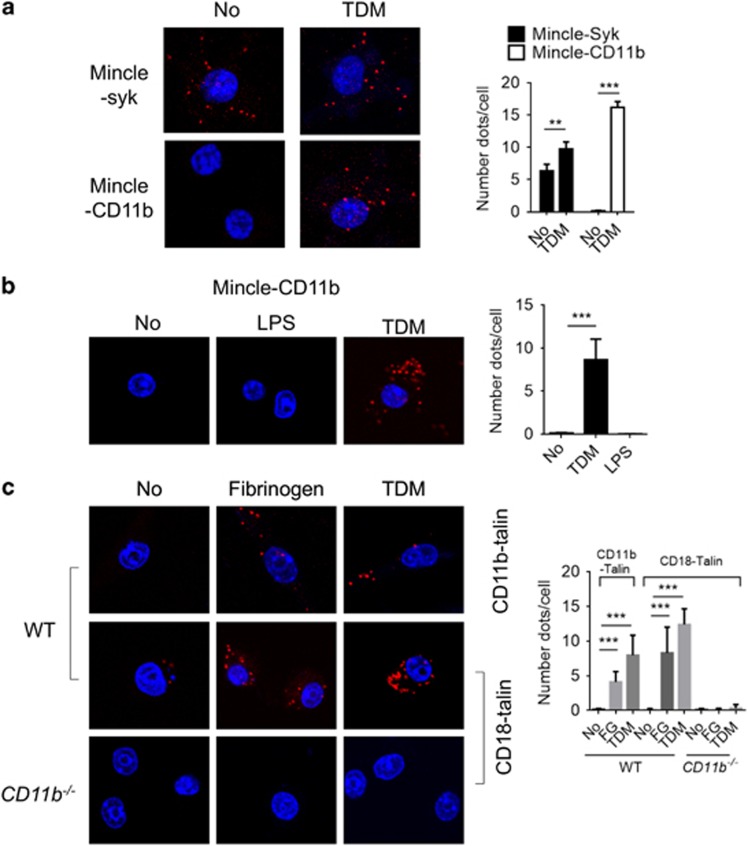
CD11b specifically interacts with Mincle upon TDM treatment. iBMM cells were transfected with the indicated tagged-plasmids. (**a**) PLA was performed for detection of Mincle-CD11b and Mincle-Syk interactions in iBMMs that were stimulated with TDM for 12 h. (**b**) The Mincle–CD11b interactions following stimulation with TDM (50 μg ml^−1^) for 12 h or LPS (100 ng ml^−1^) for 6 h were compared. (**c**) Interactions of CD11b/CD18 with endogenous talin in WT and CD11b^−/−^ iBMM cells were examined after stimulation with TDM (50 μg ml^−1^) for 24 h or fibrinogen (1 μg ml^−1^) for 30 min. Interactions were visualized as fluorescent spots (red, PLA signal), and the nuclei were stained with DAPI (blue). The number of PLA signals was determined for at least 50 cells for each condition. Data are representative of three independent experiments. **P<0.01, ***P<0.0001 (two-tailed unpaired Student’s *t*-test).

As Mincle signaling is negatively regulated by CD11b following TDM stimulation, we next examined whether CD11b directly interacts with Mincle and/or its downstream adaptor proteins and regulators. Because Syk binds FcRγ, which is the adaptor molecule for Mincle,^25^ we first examined the association between Mincle and Syk via a proximal ligation assay (PLA) in iBMMs transiently transfected with epitope-tagged Mincle and Syk. A PLA with antibodies recognizing the specific epitopes tagged to Mincle and Syk revealed a number of strong signals regardless of TDM treatment (Figure 4a). We next examined the interaction of CD11b with Mincle. Although there was no PLA signal between Mincle and CD11b under resting conditions, the number of interaction signals dramatically increased following TDM stimulation. In addition, no binding between CD11b and Mincle was observed following LPS stimulation, demonstrating that TDM specifically induced their interaction (Figure 4b).

We asked whether TDM-Mincle could also activate the CD11b integrin through an inside-out signal dependent on talin.^26^ To this end, the interaction between the CD11b/CD18 heterodimer and talin on iBMMs after treatment with fibrinogen or TDM was examined by PLA. Under basal conditions, no interaction between CD11b/CD18 and talin was observed; however, TDM treatment induced strong binding, similar to the effects of fibrinogen treatment on CD11b/CD18 and talin (Figure 4c). Intriguingly, these interactions required functional CD11b/CD18 heterodimers, as CD11b deficiency disrupted the interaction between CD18 and talin upon stimulation with TDM or fibrinogen. These results indicate that Mincle signaling triggers the binding of CD11b/CD18 and talin in macrophages, which might lead to the inside-out activation of CD11b/CD18 integrin; the combined integrin receptors may then downregulate Mincle signaling through a direct interaction.

### Activated CD11b attenuates Mincle signaling via Lyn kinase

**Figure 5:**
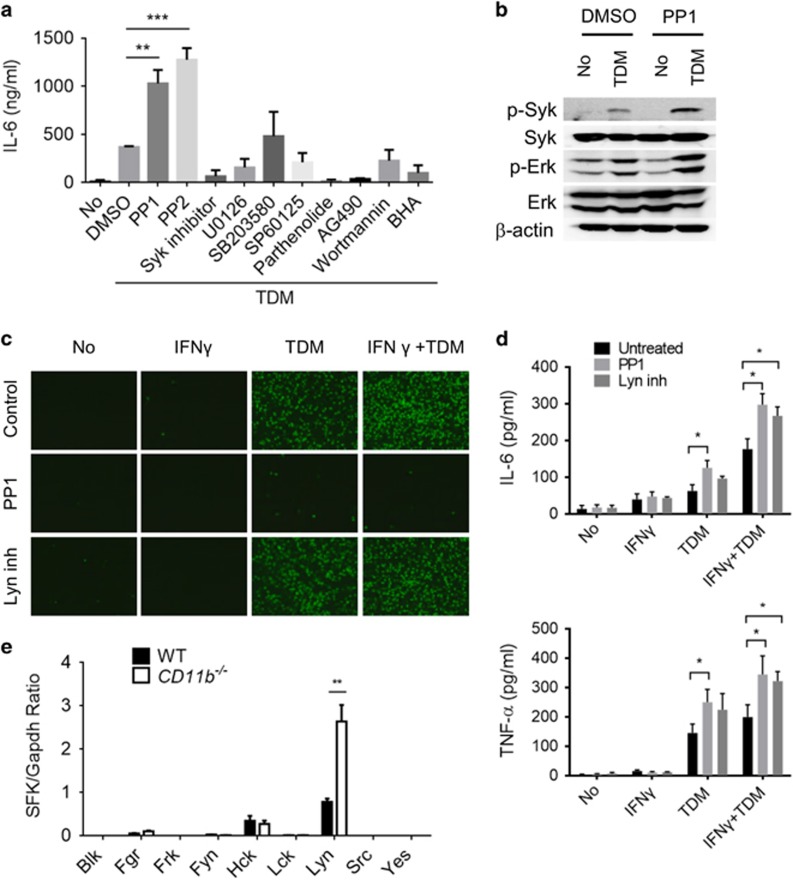
Lyn inhibits Mincle signaling by inhibiting Mincle downstream targets. (**a**) IL-6 levels in culture supernatants were measured in WT BMMs stimulated with TDM in the presence of PP1 (Src family kinase inhibitor, 5 μM), PP2 (Src family kinase inhibitor, 100 nM), Syk inhibitor (10 μM), U0126 (MEK1/2 inhibitor, 10 μM), SB203580 (p38 inhibitor, 10 μM), SP60125 (JNK inhibitor, 10 μM), Parthenolide (NFκB inhibitor, 5 μM), AG490 (JAK inhibitor, 25 μM), wortmannin (PI-3 K inhibitor, 10 μM) and BHA (antioxidant, 50 μM) after 24 h culture by ELISA. (**b**) Syk and Erk kinase activation in WT BMMs that were treated with TDM and either DMSO or PP1 (5 μM) for 6 h was analyzed by immunoblot. β-Actin protein expression was used as a loading control in immunoblot assays (**c**, **d**) WT and CD11b^−/−^ neutrophils were treated with TDM (50 μg ml^−1^), IFN-γ (10 ng ml^−1^), PP1 (5 μM) or Lyn inhibitor (2 μM), and then (**c**) attached cells were imaged with a fluorescent microscope after 6 h of treatment (× 40), and (**d**) cytokine production was measured by ELISA after 24 h of stimulation. (**e**) WT and CD11b^−/−^ BMMs were stimulated with TDM for 24 h, and the mRNA levels of Src family kinases (SFKs) were quantified by qRT-PCR. Data are representative of at least three (**a**, **c**–**e**) or two (**b**) independent experiments. *P<0.05, **P<0.01, ***P<0.0001 (two-tailed unpaired Student’s *t*-test).

To understand the regulatory role of CD11b in Mincle signaling, BMMs were treated with inhibitors of various signaling pathways and TDM-induced secretion of IL-6 was examined. Inhibitors of Syk, Erk1/2 (U0126) and IKK (parthenolide) abolished TDM-induced IL-6 cytokine production completely, demonstrating their involvement in Mincle-dependent IL-6 production. Interestingly, treatment with Src family tyrosine kinase (SFK) inhibitors (PP1 and PP2) actually increased IL-6 production after TDM stimulation, consistent with the upregulation observed in TDM-stimulated CD11b-deficient macrophages (Figure 5a). Mincle-dependent Syk and Erk1/2 phosphorylation was also highly enhanced in PP1-treated macrophages upon TDM stimulation (Figure 5b). Similarly, PP1 treatment resulted in increased IL-6 production in neutrophils but impaired cell adhesion, as described previously^12^ (Figures 5c and d). These results indicate that both Mincle-dependent cell adhesion and cytokine production are regulated by SFKs. To identify which SFKs were targeted by PP1 under the conditions described above, the expression of various SFKs was examined in TDM-treated WT and CD11b^−/−^ macrophages by qRT-PCR. Among the nine SFKs examined, *Fgr*, *Hck* and *Lyn* were induced in WT macrophages by TDM treatment, and *Lyn* was strongly increased in CD11b^−/−^ macrophages (Figure 5e). Furthermore, treatment of neutrophils with a Lyn-specific inhibitor increased their IL-6 production significantly, without impairing cell adhesion (Figures 5c and d). These results suggest that Lyn kinase is the SFK primarily mediating the inhibitory effect of CD11b signaling against Mincle-dependent proinflammatory cytokine production.

To confirm the role of *Lyn* in Mincle-mediated signaling, *Lyn* was knocked out in iBMMs using the Cas9/CRISPR system (Supplementary Figure 6a–c). Compared with WT iBMMs, Lyn^−/−^ iBMMs showed enhanced Syk and Erk1/2 phosphorylation and stronger induction of proinflammatory genes, including *Tnf*, *Il6*, *Ccl2*, *Cxcl2* and *Nos2*, upon TDM stimulation (Supplementary Figures 7 and 6). Together, these results may indicate that CD11b regulates the Mincle pathway through the Lyn kinase.

### TDM-dependent binding of Lyn with CD11b, Mincle and SHP1

**Figure 6:**
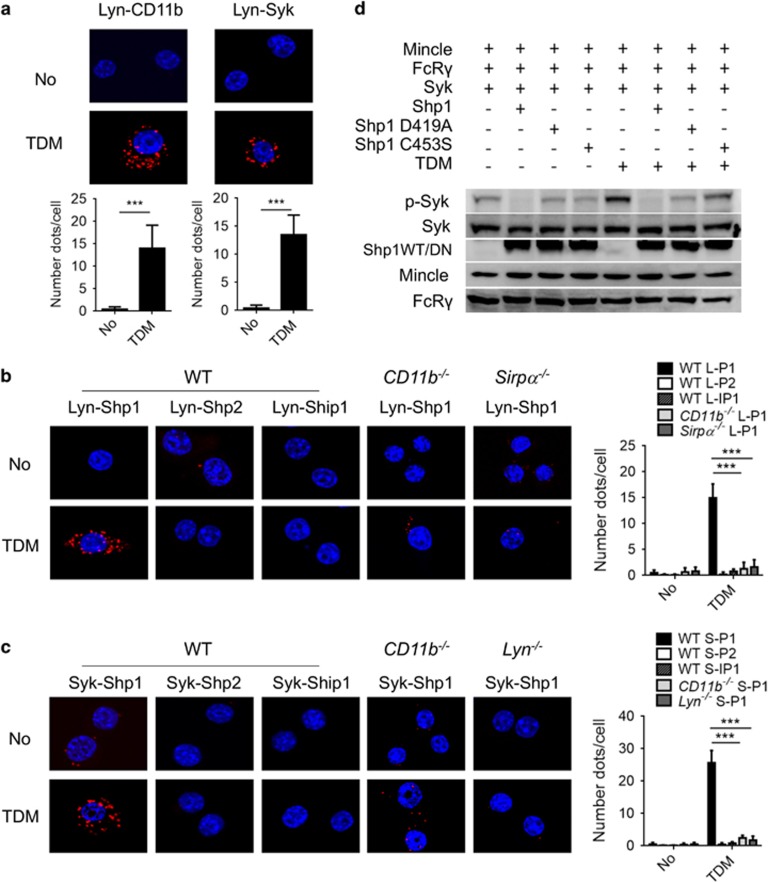
Lyn recruits SHP1 to dephosphorylate Syk. iBMMs were transfected with Flag-CD11b and HA-Lyn or HA-Lyn and Flag-Syk (**a**), HA-Lyn and Myc-SHP1, Myc-SHP2 or Myc-SHIP1 (**b**) or Flag-Syk and Myc-SHP1, Myc-SHP2 or Myc-SHIP1 (**c**) for 24 h and then stimulated with TDM for 12 h and assessed by PLA assay. (**a**) The interaction between Lyn and CD11b or Syk was measured by PLA assay. (**b**, **c**) Specific binding of Lyn and Syk to SHP1, SHP2 and SHIP1 was examined in WT and gene-deficient iBMMs. Interactions were visualized as fluorescent spots (red, PLA signal). Nuclei were stained with DAPI (blue). The number of PLA signals was determined for at least 50 cells for each condition. (**d**) Immunoblot analysis of 293T cells that were transiently transfected with V5-Mincle, Myc-FcRγ and Flag-Syk together with Flag-tagged WT or phosphatase-inactive SHP1 (C453S or D419A) for 24 h. Cells were then treated with TDM for 6 h. Data are representative of three independent experiments. ***P<0.0001 (two-tailed unpaired Student’s *t*-test).

Next, the association between Lyn and CD11b in macrophages was examined. Although no interaction was observed between CD11b and Lyn under basal conditions, TDM stimulation strongly induced this interaction (Figure 6a). Consistent with the TDM-dependent binding of Mincle to CD11b, Lyn and Syk also exhibited TDM-dependent binding to CD11b (Figure 6a). Therefore, TDM stimulation appears to induce the formation of a receptor complex that includes Mincle, CD11b and their interacting adaptors and kinases.

Previous studies revealed that the repressive role of the tyrosine kinase Lyn mainly relies on the recruitment of inhibitory phosphatases, such as SH2 domain-containing phosphatase 1 (SHP1), SHP2 and SH2 domain-containing 5’-inositol phosphatase (SHIP1).^27^ PLAs were performed before and after TDM treatment to identify the specific interacting phosphatase(s) of Lyn in response to TDM stimulation. Among the three phosphatases tested, only SHP1 interacted with Lyn and Syk specifically upon TDM treatment (Figures 6b and c). These interactions appeared to be dependent on CD11b, in that no such interaction was detected in CD11b-deficient cells. In addition, the TDM-dependent Syk-SHP1 interaction was defective in Lyn^−/−^ iBMMs. These results indicate that activated Mincle induces formation of an adaptor complex with CD11b signaling molecules.

We next tested whether the phosphatase activity of SHP1 is required for the downregulation of Mincle signaling, particularly the Syk phosphorylation. To this end, the Mincle/CD11b receptor complex was reconstituted in human embryonic kidney 293 (HEK293) cells by co-transfecting expression constructs for Mincle, FcRγ, Syk and either WT or dominant-negative SHP1 (D419A and C453S). In the reconstituted cells, TDM treatment induced Syk phosphorylation, which was diminished significantly by the addition of functional SHP1 (Figure 6d). The dominant-negative forms of SHP1, however, failed to dephosphorylate Syk. Therefore, this result indicates that SHP1 could inhibit Mincle signaling by dephosphorylating Syk. Together with the PLA assay data, these findings suggest that SHP1 is recruited by CD11b–Lyn to dephosphorylate Syk, thereby inhibiting Mincle signaling.

### SIRPα is critical for the SHP1 recruitment

**Figure 7:**
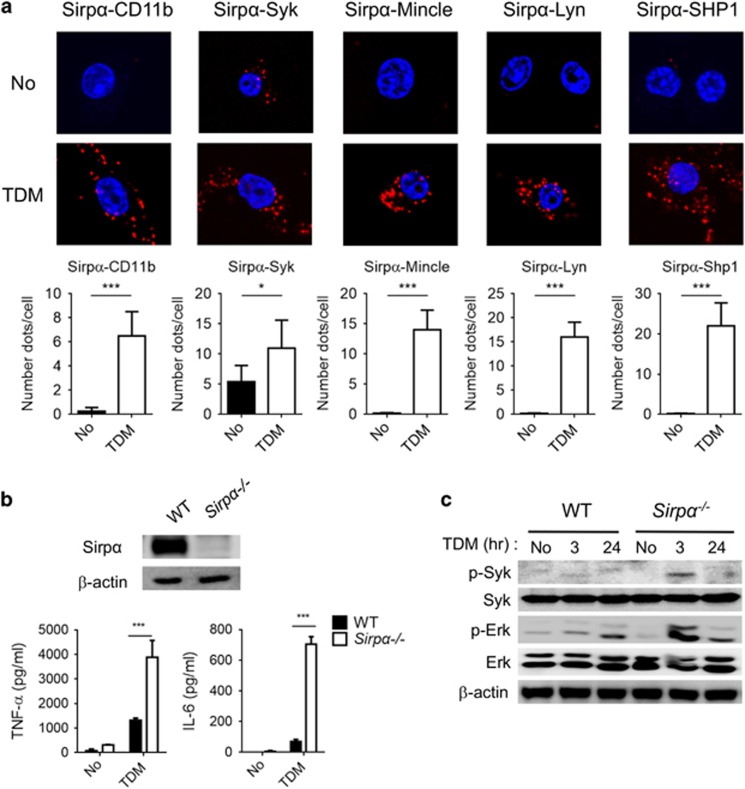
ITIM-containing SIRPα is critical for SHP1 docking and interaction with Syk. (**a**) iBMM cells were transfected with Flag-CD11b, Flag-Syk, V5-Mincle, HA-Lyn or Flag-Shp1 for 24 h and then treated with TDM for 24 h before assessment by PLA assay. Interactions between endogenous SIRPα and the transfected proteins were visualized as fluorescent spots (red, PLA signal). Nuclei were stained with DAPI (blue). The number of PLA signals was determined for at least 50 cells for each condition. (**b**) Knockdown of SIRPα was confirmed by immunoblot (top panel). Culture media was collected after 24 h of TDM stimulation and subjected to ELISA for measurement of IL-6 and TNF-α production (bottom panel). (**c**) WT and SIRPα-deficient iBMMs were stimulated with TDM for the indicated times, and activation of Syk and Erk was determined by immunoblot. β-Actin protein expression was used as a loading control. Data are representative of two (**c**) or three (**a**, **b**) independent experiments. *P<0.05, ***P<0.0001 (two-tailed unpaired Student’s *t*-test).

SHP1 phosphatase recruitment normally requires immunoreceptor tyrosine-based inhibitory motif (ITIM)-containing receptors to serve as docking sites. The two ITIM-containing receptors Pirb and SIRPα have been studied extensively in association with SHP1.^28, 29^ To determine which receptor can associate with SHP1 and Lyn upon TDM stimulation, a PLA assay was performed. Pirb exhibited strong binding with CD11b, Syk, Lyn and SHP1 independently of TDM treatment (Supplementary Figure 8); however, no interaction signal was observed for Pirb with Mincle even in the presence of TDM stimulation. These results indicate that Pirb may interact with SHP1 but that this interaction is not related to the Mincle signaling pathway. On the other hand, although SIRPα did not interact with any of the Mincle/CD11b receptor complex components tested under resting conditions, this receptor produced strong PLA signals with CD11b, Syk, Lyn, SHP1 and Mincle upon TDM stimulation (Figure 7a). In addition, binding of SIRPα to the CD11b/Mincle complex was nearly completely disrupted in Lyn^−/−^ and Syk^−/−^ iBMMs (Supplementary Figure 9). Therefore, SIRPα appears to be a member of the Mincle/CD11b receptor complex.

To investigate the requirement of SIRPα for the regulation of Mincle signaling, SIRPα-deficient iBMM cell lines were generated and evaluated for their response to TDM challenge (Figure 7b). Phosphorylation of Syk and Erk1/2 in response to TDM stimulation was increased in SIRPα^−/−^ cells than in WT cells (Figure 7c). In addition, SIRPα^−/−^ cells secreted more TNF-α and IL-6 and had stronger induction of proinflammatory genes (*Tnf*, *Il6*, *Il1*α*, Ccl2* and *Il12b*) than WT cells (Figure 7b and Supplementary Figure 6). Furthermore, the Lyn–SHP1 interaction was also disrupted in SIRPα^−/−^ iBMMs (Figure 6b), suggesting that SIRPα was required for CD11b-mediated negative regulation of Mincle signaling.

To examine the physiological relevance of the iBMM-based analysis system, rescue experiments were performed for CD11b and Syk in iBMM cell lines with deletions of the corresponding genes. CD11b-deficient iBMMs showed enhanced Mincle-dependent IL-6 production, as did the CD11b^−/−^ BMMs, while transfection with Flag-tagged CD11b reduced the Mincle signal. In addition, Mincle signaling was almost completely abrogated in the Syk-deficient iBMMs, but transfection with Flag-tagged Syk rescued Mincle-dependent IL-6 production in those cells (Supplementary Figures 10a and b).

### CD11b initiates the formation of an inhibitory complex to bind Mincle

**Figure 8:**
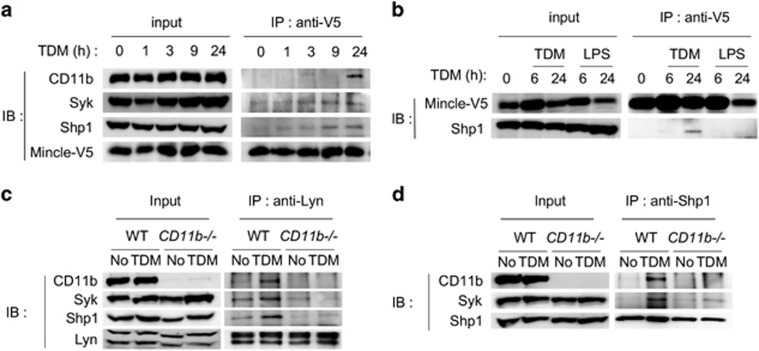
Binding of CD11b and Mincle upon TDM stimulation. (**a**, **b**) iBMM cells stably expressing Mincle were stimulated with TDM or LPS. Lysates were immunoprecipitated with an anti-Mincle Ab, and the immunoprecipitates were probed for Mincle-V5, CD11b, Syk and SHP1 by immunoblot. (**c**, **d**) Co-immunoprecipitation of endogenous Lyn and SHP1 in WT and CD11b^−/−^ BMMs after TDM stimulation. Data are representative of at least two independent experiments.

To confirm the physical interaction between CD11b and Mincle, a co-immunoprecipitation assay was performed using iBMMs expressing an epitope-tagged Mincle. Immunoprecipitation of Mincle did not pull down CD11b in the absence of TDM treatment. However, the amount of CD11b, Syk and SHP1 that co-immunoprecipitated with Mincle gradually increased after TDM stimulation (Figure 8a). These interactions appear specific to TDM stimulation, as no such binding was observed when LPS was used instead of TDM (Figure 8b). The interactions among the signaling molecules were further validated using endogenous proteins. Pull-down of Lyn in WT macrophages revealed a TDM-dependent complex containing Lyn, CD11b, SHP1 and Syk that was disrupted in CD11b-deficient macrophages (Figure 8c and Supplementary Figure 11a). Moreover, immunoprecipitation with an anti-SHP1 antibody also revealed a TDM- and CD11b-dependent interaction among CD11b, Syk and SHP1 (Figure 8d and Supplementary Figure 11b). Taken together with the PLA results, these data confirm the formation of an inhibitory complex that contains Lyn, Syk and SHP1 and binds CD11b and Mincle.

### The Lyn activator MLR1023 suppresses Mincle signaling

**Figure 9:**
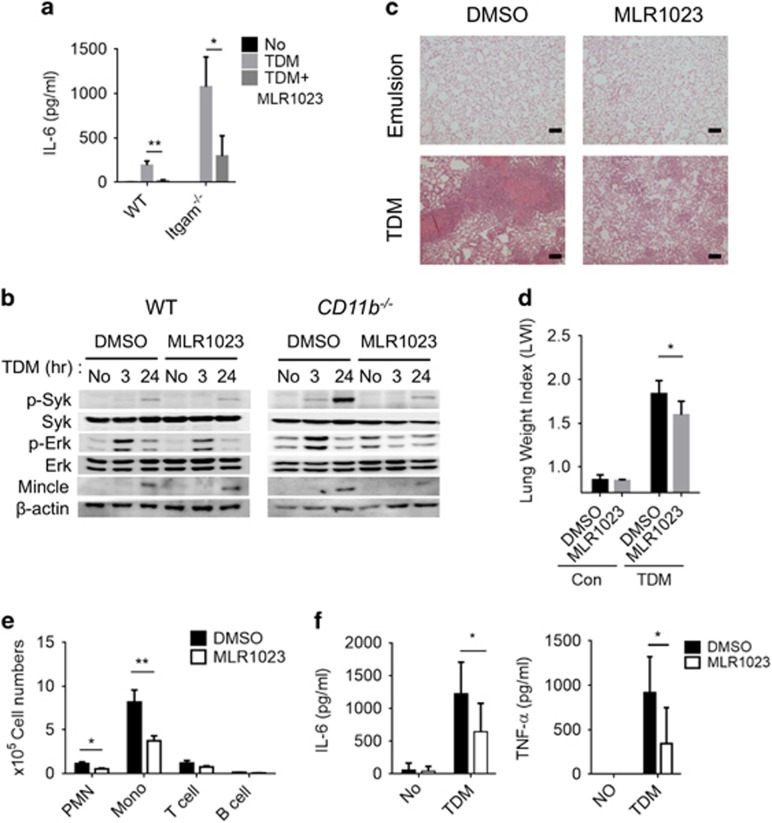
The Lyn activator MLR1023 inhibits TDM signaling both *in vivo* and *in vitro*. (**a**) Production of IL-6 by WT and CD11b^−/−^ BMMs challenged with TDM and MLR1023 (1 ng ml^−1^) or DMSO for 24 h was assessed by ELISA. (**b**) Western blotting assay of Syk and Erk kinase activity and Mincle induction in WT and CD11b^−/−^ BMMs treated with TDM and MLR1023 (1 ng ml^−1^) or DMSO for 24 h. β-Actin protein expression was used as a loading control. Experimental and control mice (*n*=8 mice per group) were intravenously injected with 3.75 mg kg^−1^ TDM in an oil-in-water emulsion on day 0. Then the experimental mice were intraperitoneally injected with MLR1023 (6 mg kg^−1^ in PBS) every day beginning on day 1, until the mice were killed 7 days post-TDM challenge. The control mice were injected with 1% DMSO in PBS. (**c**) Lung tissues were isolated and stained with hematoxylin and eosin (H&E) for histology analysis after the lung weight index (LWI) (**d**) was determined. (**e**) Leukocyte subsets were analyzed by flow cytometry using distinct markers for monocytes and macrophages (Mo/Ma; CD11b+ Ly6G−), neutrophils (PMN; CD11b+ Ly6G+), T cells (CD3+) and B cells (CD19+). (**f**) Lung homogenates were analyzed by ELISA for TNF-α and IL-6. Data are representative of two independent experiments. (**c**–**f**, mean and s.d. of eight mice per group.) *P<0.05, **P<0.01 (two-tailed unpaired Student’s *t*-test).

Because Lyn has a pivotal role in the CD11b-mediated negative regulation of Mincle signaling, the ability of the Lyn kinase activator MLR1023 to enhance anti-Mincle activity was examined. Contrary to the stimulatory effect of PP1 on TDM-induced proinflammatory cytokine production (Figure 5a), treatment with MLR1023 arrested TDM-induced IL-6 production in WT macrophages and restored the hyperinflammatory response of CD11b^−/−^ macrophages to levels similar to those seen in WT macrophages (Figure 9a). Furthermore, MLR1023 treatment reduced the TDM-dependent phosphorylation of Syk and Erk1/2 in both WT and CD11b^−/−^ BMMs (Figure 9b). We found that activation of Lyn using MLR1023 reversed the effect of CD11b deficiency on the Lyn–Shp1 and Syk–Shp1 interactions (Supplementary Figure 12a). These results confirmed the negative regulatory role of Lyn in Mincle signaling.

To examine the physiological relevance of CD11b-mediated inhibition of Mincle-dependent inflammation, the effect of enhanced CD11b signaling on TDM-induced granuloma formation in mice was investigated. The Lyn activator MLR1023 was administered to TDM-challenged mice and the effects on TDM-induced granuloma formation were examined. Compared with the control group, MLR1023-treated mice exhibited less severe TDM-induced lung granulomas and lung swelling (Figures 9c and d). Leukocyte recruitment to the lung (Figure 9e) and TNF-α and IL-6 production (Figure 9f) were also decreased in the MLR1023-treated mice, indicating that MLR1023 suppresses TDM-induced inflammation. Intriguingly, MLR1023 treatment also decreased the TDM-induced granuloma response in CD11b^−/−^ mice (Supplementary Figures 12b–d), confirming the epistatic effect of Lyn in the CD11b signaling pathway. Therefore, CD11b signaling has an important inhibitory role in the regulation of Mincle-dependent inflammatory responses against mycobacterial infection. These data suggest that the Lyn kinase may be an effective target for the treatment of the excessive inflammatory response caused by this infection.

## Discussion

Mincle has a central role in the host defense against mycobacterial infection as the major receptor for mycobacterial cell wall component TDM. The activating role of this signaling molecule in the proinflammatory response has been well studied; however, little is known about the mechanism that leads to dampening of this inflammatory signal. In this study, we provide multiple lines of evidence demonstrating that CD11b is the crucial negative regulator of Mincle signaling and therefore has an important role in Mtb infection.

First of all, our observation that CD11b deficiency resulted in a significantly enhanced Mincle-dependent inflammatory response against TDM and BCG challenge extends the function of CD11b in Mtb infection. Phagocytes from patients with tuberculosis possess augmented CD11b/CD18 expression, which is thought to promote cell adhesion and accumulation at the infection site.^30^ During Mtb infection, the immune response is mediated by TLRs, which are activated by various molecular patterns on Mtb.^31^ Recent work revealed that CD11b facilitates proteasomal degradation of Myd88 and TRIF upon TLR3, 4 and 9 activation, thereby inhibiting the inflammatory response.^14^ Bang and colleagues also reported that CD11b downregulates DC-mediated cross-priming through miR-146a.^32^ Here we demonstrated that CD11b interferes with the proinflammatory response induced by the Mtb-specific PAMP TDM and confirmed that live Mtb infection also induces increased cytokine production in CD11b^−/−^ BMMs. Although TLR and Mincle signaling are negatively regulated by CD11b through different mechanisms, these signals converge on nuclear transcription factor-kappaB and synergistically enhance the inflammatory response toward Mtb infection.^33, 34^ Altogether, CD11b appears to have a broad role in the negative regulation of the proinflammatory response toward PAMPs expressed by Mtb.

Moreover, CD11b may be involved in inflammatory responses toward Mtb infection via another Mincle ligand. During the late phase of Mtb infection, infected macrophages are cleared by apoptosis, necrosis and autophagy.^35^ Necrotic cell death results in the release of spliceosome-associated protein 130 (SAP130), a soluble component of the U2 small nuclear ribonucleoprotein-associated protein that is normally found in the nucleus.^36^ Yamasaki *et al.*^25^ reported that ligation of SAP130 to Mincle on macrophages also elicits proinflammatory responses through FcRγ and Card9, similar to the effects of TDM stimulation. Hence, these findings indicate that CD11b may further regulate the immune response during the late stage of Mtb infection through SAP130-mediated Mincle signaling.

Second, our results indicate that negative regulation of Mincle signaling by CD11b occurs via the Lyn kinase. As an SFK, Lyn has both positive and negative roles in modulating immune function and is also associated with integrin-mediated cellular homeostasis.^37^ Lyn provides a positive signal via phosphorylation of ITAM-carrying proteins, such as FcRγ, to recruit Syk and activate Syk-mediated signaling.^38^ Conversely, Lyn can downregulate signaling via direct interactions with a target protein such as interferon regulatory factor 5 (IRF5). Lyn ultimately inhibits IRF5 ubiquitination and phosphorylation, impairing the IRF5-mediated TLR–Myd88 signal.^39^ Meanwhile, the main negative regulatory role of Lyn is dependent on the recruitment of phosphatases such as SHP1, SHP2 and SHIP1, which target proteins through ITIM domain-carrying receptors, such as SIRPα and Pirb. Here we found that Lyn interacts with CD11b in a Mincle signaling-dependent manner. Following inhibition of Lyn with PP1 in BMMs or deletion of Lyn in iBMMs, we observed a hyper-response toward TDM stimulation similar to that observed in CD11b^−/−^ cells following TDM stimulation. The negative regulatory role of Lyn has been thoroughly investigated within the context of B-cell receptor signaling and integrin-mediated adhesion. Recent work defined CD11b as the major modulator in the formation of the inhibitory complex Lyn–CD22–SHP1, which restrains B-cell receptor signals.^16^ In addition, Lyn^−/−^ macrophages display decreased activation of SIRPα, Pirb and SHP1 upon growth factor stimulation, suggesting that Lyn, SIRPα/Pirb and SHP1 may function together to exert negative regulatory effects in response to growth factors.^40^ Similar to these findings, we found that Lyn dephosphorylates Syk, a target previously known to be positively regulated by Lyn. This dephosphorylation was mediated by Lyn’s recruitment of SHP1 to the docking protein SIRPα in a CD11b-dependent manner.

Treatment of cells with PP1 during TDM stimulation revealed a broad role for SKFs in Mincle signaling. Integrin activation can be more easily studied in neutrophils than in macrophages, as robust functional readouts exist for these cells.^41^ Upon integrin activation, neutrophils show greatly enhanced adhesion. TDM stimulation facilitated adhesion in neutrophils, which was abrogated by PP1 treatment or CD11b deficiency but not by inhibition of Lyn. These results suggest that CD11b has a major role in TDM-induced neutrophil adhesion, while SFKs besides Lyn could positively modulate this process. On the other hand, utilizing a Lyn-specific inhibitor led to enhanced cytokine production in neutrophils, with no influence on cell adhesion. These findings are consistent with our predicted model, in which Lyn is critical for the formation of an inhibitory complex that is dependent on signals transduced by activated CD11b but does not affect CD11b-mediated adhesion induced by TDM.

In addition, treatment with the Lyn activator MLR1023 inhibited Mincle signaling both *in vitro* and *in vivo*. MLR1023 is a selective Lyn activator and a potential insulin sensitizer that was developed as a candidate therapeutic drug for type II diabetes.^42^ Interestingly, enhanced Mincle expression was detected in obesity-induced adipose tissue fibrosis, suggesting that augmented Mincle signaling may contribute to adipose tissue fibrosis formation and thereby promote obesity.^43^ Fibrosis is a similar process to granuloma formation and involves the accumulation of activated macrophages that highly express CD11b.^44, 45^ In our granuloma model, treatment of TDM-challenged mice with MLR1023 decreased Mincle signaling and inhibited granuloma formation. Therefore, MLR1023 treatment of obese mice may also decrease adipose fibrosis formation by inhibiting Mincle signaling. Recently, Lee *et al.*^46^ found that enhanced Mincle signaling is strongly correlated with uveitis, an autoimmune disease of the eye. Thus further studies might be warranted to determine whether treatment with MLR1023 could inhibit Mincle signaling in these Mincle-related diseases.

Finally, regulation of CD11b in TDM–Mincle signaling specifically affects macrophages and neutrophils, but not DCs. Although CD11b is as highly expressed in DCs as in macrophages,^47^ its function might vary in different cell types, even in response to the same stimulation. For example, in the context of LPS stimulation, CD11b exerts a negative role in macrophages by facilitating the degradation of Myd88 and TRIF^14^ but positively influences LPS signaling in DCs.^21^ Meanwhile, SHP1 has also been reported to form an inhibitory axis following activation by Mincle signaling in DCs; this results in reduced adaptive immunity to *Leishmania major* infection.^48^ However, in that paper, trehalose-6,6-dibehenate was not the inducer of the Mincle–SHP1 interaction in DCs. Therefore, the Lyn–SIRPα–SHP1 inhibitory complex may be formed specifically in response to Mincle signaling in macrophages and neutrophils.

Similarly, Lyn and SIRPα were observed to have opposing effects in deficient iBMMs, which might be explained by the multiple biological functions of Lyn and SIRPα. Lyn is particularly well known as a dual functional regulator in macrophages, where it negatively regulates signaling induced by growth factors and integrin activation.^37^ Some studies have also implicated Lyn activation particularly in signaling through the IL-3 and IL-6 receptors.^49^ Meanwhile, SIRPα can bind to and be activated by the transmembrane protein CD47, which is expressed on immortalized cell lines such as the iBMMs that were used in this study.^50^ SIRPα–CD47 binding can induce a variety of signaling pathways that result in the inhibition of phagocytosis.^51^ Also, SIRPα expressed on macrophages can attenuate mitogen-activated protein kinase signaling and nuclear transcription factor-kappaB activation in response to LPS treatment through an association with SHP2.^52^ Considering that both Lyn and SIRPα could specifically regulate the production of distinct cytokines via divergent pathways, a deficiency in either Lyn or SIRPα may have a distinct influence on the major inhibitory function of CD11b–SHP1 in Mincle signaling.

In conclusion, we demonstrated that activation of CD11b in response to TDM acts as a critical negative regulator of Mincle signaling by promoting formation of a Lyn–SIRPα–SHP1 complex that dephosphorylates Syk. CD11b deficiency led to a hyper-response against TDM challenge, while Lyn activation by MLR1023 exerted the opposite effect and impaired the TDM-induced inflammatory response. Our results provide insight into the mechanisms involved in fine-tuning Mincle signaling during the inflammatory response and suggest Lyn as a potential target for modulation of the immune response during Mtb infection.

## References

[bib1] Cosma CL, Sherman DR, Ramakrishnan L. The secret lives of the pathogenic mycobacteria. Annu Rev Microbiol 2003; 57: 641–676. 14527294 10.1146/annurev.micro.57.030502.091033

[bib2] Davis JM, Ramakrishnan L. The role of the granuloma in expansion and dissemination of early tuberculous infection. Cell 2009; 136: 37–49. 19135887 10.1016/j.cell.2008.11.014 PMC3134310

[bib3] Ramakrishnan L. Revisiting the role of the granuloma in tuberculosis. Nat Rev Immunol 2012; 12: 352–366. 22517424 10.1038/nri3211

[bib4] Saunders BM, Cooper AM. Restraining mycobacteria: role of granulomas in mycobacterial infections. Immunol Cell Biol 2000; 78: 334–341. 10947857 10.1046/j.1440-1711.2000.00933.x

[bib5] Sasindran SJ, Torrelles JB. *Mycobacterium tuberculosis* infection and inflammation: what is beneficial for the host and for the bacterium? Front Microbiol 2011; 2: 2. 21687401 10.3389/fmicb.2011.00002 PMC3109289

[bib6] Song C, Luo L, Lei Z, Li B, Liang Z, Liu G et al. IL-17-producing alveolar macrophages mediate allergic lung inflammation related to asthma. J Immunol 2008; 181: 6117–6124. 18941201 10.4049/jimmunol.181.9.6117

[bib7] Cavalcanti YV, Brelaz MC, Neves JK, Ferraz JC, Pereira VR. Role of TNF-alpha, IFN-gamma, and IL-10 in the development of pulmonary tuberculosis. Pulm Med 2012; 2012: 745483. 23251798 10.1155/2012/745483 PMC3515941

[bib8] Redford PS, Murray PJ, O'Garra A. The role of IL-10 in immune regulation during *M. tuberculosis* infection. Mucosal Immunol 2011; 4: 261–270. 21451501 10.1038/mi.2011.7

[bib9] Baba T, Natsuhara Y, Kaneda K, Yano I. Granuloma formation activity and mycolic acid composition of mycobacterial cord factor. Cell Mol Life Sci 1997; 53: 227–232. 9104485 10.1007/PL00000595 PMC11147429

[bib10] Ishikawa E, Ishikawa T, Morita YS, Toyonaga K, Yamada H, Takeuchi O et al. Direct recognition of the mycobacterial glycolipid, trehalose dimycolate, by C-type lectin Mincle. J Exp Med 2009; 206: 2879–2888. 20008526 10.1084/jem.20091750 PMC2806462

[bib11] Lang R. Recognition of the mycobacterial cord factor by Mincle: relevance for granuloma formation and resistance to tuberculosis. Front Immunol 2013; 4: 5. 23355839 10.3389/fimmu.2013.00005 PMC3553576

[bib12] Lee WB, Kang JS, Yan JJ, Lee MS, Jeon BY, Cho SN et al. Neutrophils promote mycobacterial trehalose dimycolate-induced lung inflammation via the Mincle pathway. PLoS Pathog 2012; 8: e1002614. 22496642 10.1371/journal.ppat.1002614 PMC3320589

[bib13] Luo BH, Carman CV, Springer TA. Structural basis of integrin regulation and signaling. Annu Rev Immunol 2007; 25: 619–647. 17201681 10.1146/annurev.immunol.25.022106.141618 PMC1952532

[bib14] Han C, Jin J, Xu S, Liu H, Li N, Cao X. Integrin CD11b negatively regulates TLR-triggered inflammatory responses by activating Syk and promoting degradation of MyD88 and TRIF via Cbl-b. Nat Immunol 2010; 11: 734–742. 20639876 10.1038/ni.1908

[bib15] Ehirchiou D, Xiong Y, Xu G, Chen W, Shi Y, Zhang L. CD11b facilitates the development of peripheral tolerance by suppressing Th17 differentiation. J Exp Med 2007; 204: 1519–1524. 17562817 10.1084/jem.20062292 PMC2118631

[bib16] Ding C, Ma Y, Chen X, Liu M, Cai Y, Hu X et al. Integrin CD11b negatively regulates BCR signalling to maintain autoreactive B cell tolerance. Nat Commun 2013; 4: 2813. 24264377 10.1038/ncomms3813

[bib17] Green LC, Wagner DA, Glogowski J, Skipper PL, Wishnok JS, Tannenbaum SR. Analysis of nitrate, nitrite, and [15N]nitrate in biological fluids. Anal Biochem 1982; 126: 131–138. 7181105 10.1016/0003-2697(82)90118-x

[bib18] Yarkoni E, Rapp HJ. Granuloma formation in lungs of mice after intravenous administration of emulsified trehalose-6,6'-dimycolate (cord factor): reaction intensity depends on size distribution of the oil droplets. Infect Immun 1977; 18: 552–554. 924683 10.1128/iai.18.2.552-554.1977 PMC421268

[bib19] Perez RL, Roman J, Roser S, Little C, Olsen M, Indrigo J et al. Cytokine message and protein expression during lung granuloma formation and resolution induced by the mycobacterial cord factor trehalose-6,6'-dimycolate. J Interferon Cytokine Res 2000; 20: 795–804. 11032399 10.1089/10799900050151067

[bib20] Fossati-Jimack L, Ling GS, Cortini A, Szajna M, Malik TH, McDonald JU et al. Phagocytosis is the main CR3-mediated function affected by the lupus-associated variant of CD11b in human myeloid cells. PLoS ONE 2013; 8: e57082. 23451151 10.1371/journal.pone.0057082 PMC3579793

[bib21] Ling GS, Bennett J, Woollard KJ, Szajna M, Fossati-Jimack L, Taylor PR et al. Integrin CD11b positively regulates TLR4-induced signalling pathways in dendritic cells but not in macrophages. Nat Commun 2014; 5: 3039. 24423728 10.1038/ncomms4039 PMC3905776

[bib22] Lee WB, Kang JS, Choi WY, Zhang Q, Kim CH, Choi UY et al. Mincle-mediated translational regulation is required for strong nitric oxide production and inflammation resolution. Nat Commun 2016; 7: 11322. 27089465 10.1038/ncomms11322 PMC4837483

[bib23] Schoenen H, Huber A, Sonda N, Zimmermann S, Jantsch J, Lepenies B et al. Differential control of Mincle-dependent cord factor recognition and macrophage responses by the transcription factors C/EBPbeta and HIF1alpha. J Immunol 2014; 193: 3664–3675. 25156364 10.4049/jimmunol.1301593

[bib24] Sakaguchi I, Tsujimura M, Ikeda N, Minamino M, Kato Y, Watabe K et al. Granulomatous tissue formation of shikon and shikonin by air pouch method. Biol Pharm Bull 2001; 24: 650–655. 11411553 10.1248/bpb.24.650

[bib25] Yamasaki S, Ishikawa E, Sakuma M, Hara H, Ogata K, Saito T. Mincle is an ITAM-coupled activating receptor that senses damaged cells. Nat Immunol 2008; 9: 1179–1188. 18776906 10.1038/ni.1651

[bib26] Zhang Y, Wang H. Integrin signalling and function in immune cells. Immunology 2012; 135: 268–275. 22211918 10.1111/j.1365-2567.2011.03549.x PMC3372743

[bib27] Harder KW, Quilici C, Naik E, Inglese M, Kountouri N, Turner A et al. Perturbed myelo/erythropoiesis in Lyn-deficient mice is similar to that in mice lacking the inhibitory phosphatases SHP-1 and SHIP-1. Blood 2004; 104: 3901–3910. 15339845 10.1182/blood-2003-12-4396

[bib28] Takai T, Ono M. Activating and inhibitory nature of the murine paired immunoglobulin-like receptor family. Immunol Rev 2001; 181: 215–222. 11513143 10.1034/j.1600-065x.2001.1810118.x

[bib29] Barclay AN, Brown MH. The SIRP family of receptors and immune regulation. Nat Rev Immunol 2006; 6: 457–464. 16691243 10.1038/nri1859

[bib30] Yassin RJ, Hamblin AS. Altered expression of CD11/CD18 on the peripheral blood phagocytes of patients with tuberculosis. Clin Exp Immunol 1994; 97: 120–125. 7518366 10.1111/j.1365-2249.1994.tb06589.x PMC1534774

[bib31] Basu J, Shin DM, Jo EK. Mycobacterial signaling through toll-like receptors. Front Cell Infect Microbiol 2012; 2: 145. 23189273 10.3389/fcimb.2012.00145 PMC3504976

[bib32] Bai Y, Qian C, Qian L, Ma F, Hou J, Chen Y et al. Integrin CD11b negatively regulates TLR9-triggered dendritic cell cross-priming by upregulating microRNA-146a. J Immunol 2012; 188: 5293–5302. 22551553 10.4049/jimmunol.1102371

[bib33] Kawai T, Akira S. Signaling to NF-kappaB by Toll-like receptors. Trends Mol Med 2007; 13: 460–469. 18029230 10.1016/j.molmed.2007.09.002

[bib34] Schoenen H, Bodendorfer B, Hitchens K, Manzanero S, Werninghaus K, Nimmerjahn F et al. Cutting edge: Mincle is essential for recognition and adjuvanticity of the mycobacterial cord factor and its synthetic analog trehalose-dibehenate. J Immunol 2010; 184: 2756–2760. 20164423 10.4049/jimmunol.0904013 PMC3442336

[bib35] Xu G, Wang J, Gao GF, Liu CH. Insights into battles between *Mycobacterium tuberculosis* and macrophages. Protein Cell 2014; 5: 728–736. 24938416 10.1007/s13238-014-0077-5 PMC4180456

[bib36] Das BK, Xia L, Palandjian L, Gozani O, Chyung Y, Reed R. Characterization of a protein complex containing spliceosomal proteins SAPs 49, 130, 145, and 155. Mol Cell Biol 1999; 19: 6796–6802. 10490618 10.1128/mcb.19.10.6796 PMC84676

[bib37] Scapini P, Pereira S, Zhang H, Lowell CA. Multiple roles of Lyn kinase in myeloid cell signaling and function. Immunol Rev 2009; 228: 23–40. 19290919 10.1111/j.1600-065X.2008.00758.x PMC3248569

[bib38] Campbell KS. Signal transduction from the B cell antigen-receptor. Curr Opin Immunol 1999; 11: 256–264. 10375554 10.1016/s0952-7915(99)80042-9

[bib39] Ban T, Sato GR, Nishiyama A, Akiyama A, Takasuna M, Umehara M et al. Lyn kinase suppresses the transcriptional activity of IRF5 in the TLR-MyD88 pathway to restrain the development of autoimmunity. Immunity 2016; 45: 319–332. 27521268 10.1016/j.immuni.2016.07.015

[bib40] Harder KW, Parsons LM, Armes J, Evans N, Kountouri N, Clark R et al. Gain- and loss-of-function Lyn mutant mice define a critical inhibitory role for Lyn in the myeloid lineage. Immunity 2001; 15: 603–615. 11672542 10.1016/s1074-7613(01)00208-4

[bib41] Abram CL, Lowell CA. The diverse functions of Src family kinases in macrophages. Front Biosci 2008; 13: 4426–4450. 18508521 10.2741/3015 PMC4457449

[bib42] Saporito MS, Ochman AR, Lipinski CA, Handler JA, Reaume AG. MLR-1023 is a potent and selective allosteric activator of Lyn kinase *in vitro* that improves glucose tolerance *in vivo*. J Pharmacol Exp Ther 2012; 342: 15–22. 22473614 10.1124/jpet.112.192096

[bib43] Tanaka M, Ikeda K, Suganami T, Komiya C, Ochi K, Shirakawa I et al. Macrophage-inducible C-type lectin underlies obesity-induced adipose tissue fibrosis. Nat Commun 2014; 5: 4982. 25236782 10.1038/ncomms5982

[bib44] Stanton MC, Chen SC, Jackson JV, Rojas-Triana A, Kinsley D, Cui L et al. Inflammatory signals shift from adipose to liver during high fat feeding and influence the development of steatohepatitis in mice. J Inflamm (Lond) 2011; 8: 8. 21410952 10.1186/1476-9255-8-8 PMC3070617

[bib45] Sun K, Tordjman J, Clement K, Scherer PE. Fibrosis and adipose tissue dysfunction. Cell Metab 2013; 18: 470–477. 23954640 10.1016/j.cmet.2013.06.016 PMC3795900

[bib46] Lee EJ, Brown BR, Vance EE, Snow PE, Silver PB, Heinrichs D et al. Mincle activation and the Syk/Card9 signaling axis are central to the development of autoimmune disease of the eye. J Immunol 2016; 196: 3148–3158. 26921309 10.4049/jimmunol.1502355 PMC4799727

[bib47] Merad M, Sathe P, Helft J, Miller J, Mortha A. The dendritic cell lineage: ontogeny and function of dendritic cells and their subsets in the steady state and the inflamed setting. Annu Rev Immunol 2013; 31: 563–604. 23516985 10.1146/annurev-immunol-020711-074950 PMC3853342

[bib48] Iborra S, Martinez-Lopez M, Cueto FJ, Conde-Garrosa R, Del Fresno C, Izquierdo HM et al. Leishmania uses mincle to target an inhibitory ITAM signaling pathway in dendritic cells that dampens adaptive immunity to infection. Immunity 2016; 45: 788–801. 27742545 10.1016/j.immuni.2016.09.012 PMC5074365

[bib49] Torigoe T, O'Connor R, Santoli D, Reed JC. Interleukin-3 regulates the activity of the LYN protein-tyrosine kinase in myeloid-committed leukemic cell lines. Blood 1992; 80: 617–624. 1638019

[bib50] Willingham SB, Volkmer JP, Gentles AJ, Sahoo D, Dalerba P, Mitra SS et al. The CD47-signal regulatory protein alpha (SIRPa) interaction is a therapeutic target for human solid tumors. Proc Natl Acad Sci USA 2012; 109: 6662–6667. 22451913 10.1073/pnas.1121623109 PMC3340046

[bib51] Matozaki T, Murata Y, Okazawa H, Ohnishi H. Functions and molecular mechanisms of the CD47-SIRPalpha signalling pathway. Trends Cell Biol 2009; 19: 72–80. 19144521 10.1016/j.tcb.2008.12.001

[bib52] Kong XN, Yan HX, Chen L, Dong LW, Yang W, Liu Q et al. LPS-induced down-regulation of signal regulatory protein {alpha} contributes to innate immune activation in macrophages. J Exp Med 2007; 204: 2719–2731. 17954568 10.1084/jem.20062611 PMC2118489

