# Supplementary material for: Integrin CD11b negatively regulates Mincle-induced signaling via the Lyn–SIRPα–SHP1 complex
Source: Exp Mol Med. 2018 Feb 5;50(2):e439–. doi: 10.1038/emm.2017.256 (PMC5992981; doi:10.1038/emm.2017.256)
Supplement: Supplementary Figures and Tables [file emm2017256x1.pdf]

S Fig 1

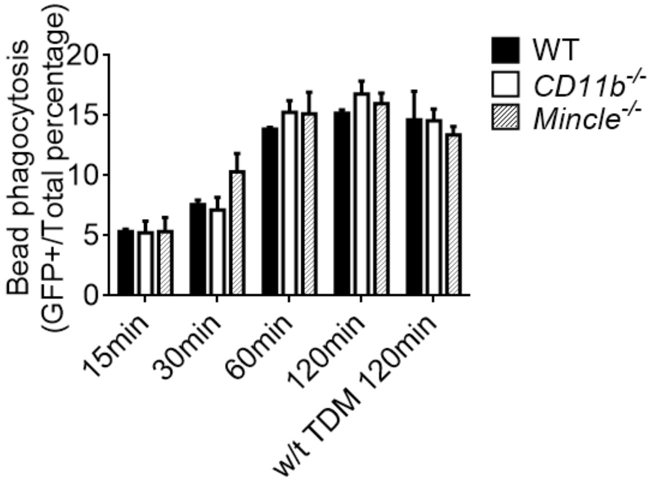

Bead phagocytosis
(GFP+/Total percentage)
0
5
10
15
20
15min
30min
60min
120min
w/t TDM 120min
WT
CD11b-/-
Mincle-/-

S Fig 2

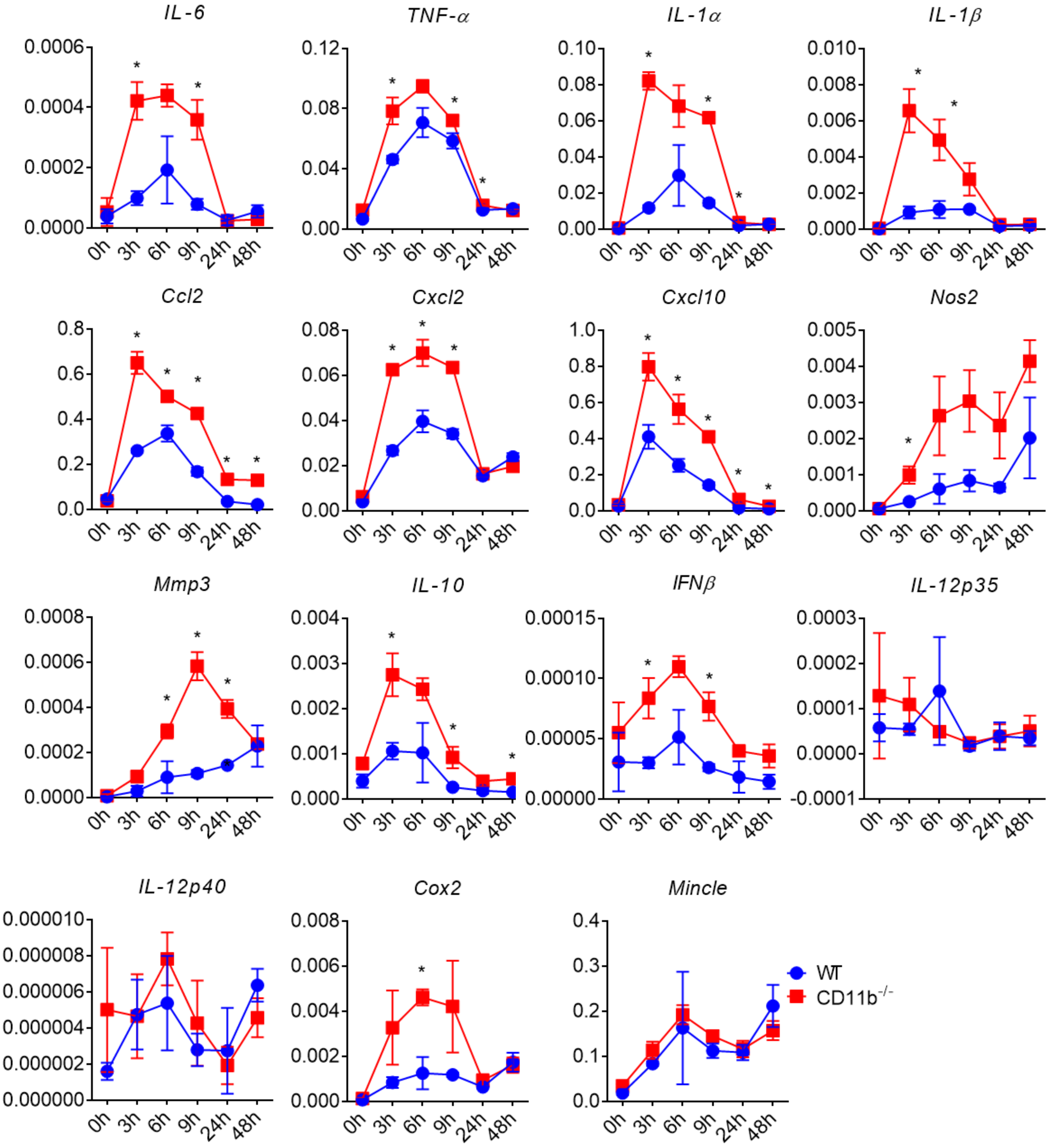
IL-6
TNF-α
IL-1α
IL-1β
Ccl2
Cxcl2
Cxcl10
Nos2
Mmp3
IL-10
IFNβ
IL-12p35
IL-12p40
Cox2
Mincle
0h 3h 6h 9h 24h 48h
WT
CD11b-/-

S Fig 3

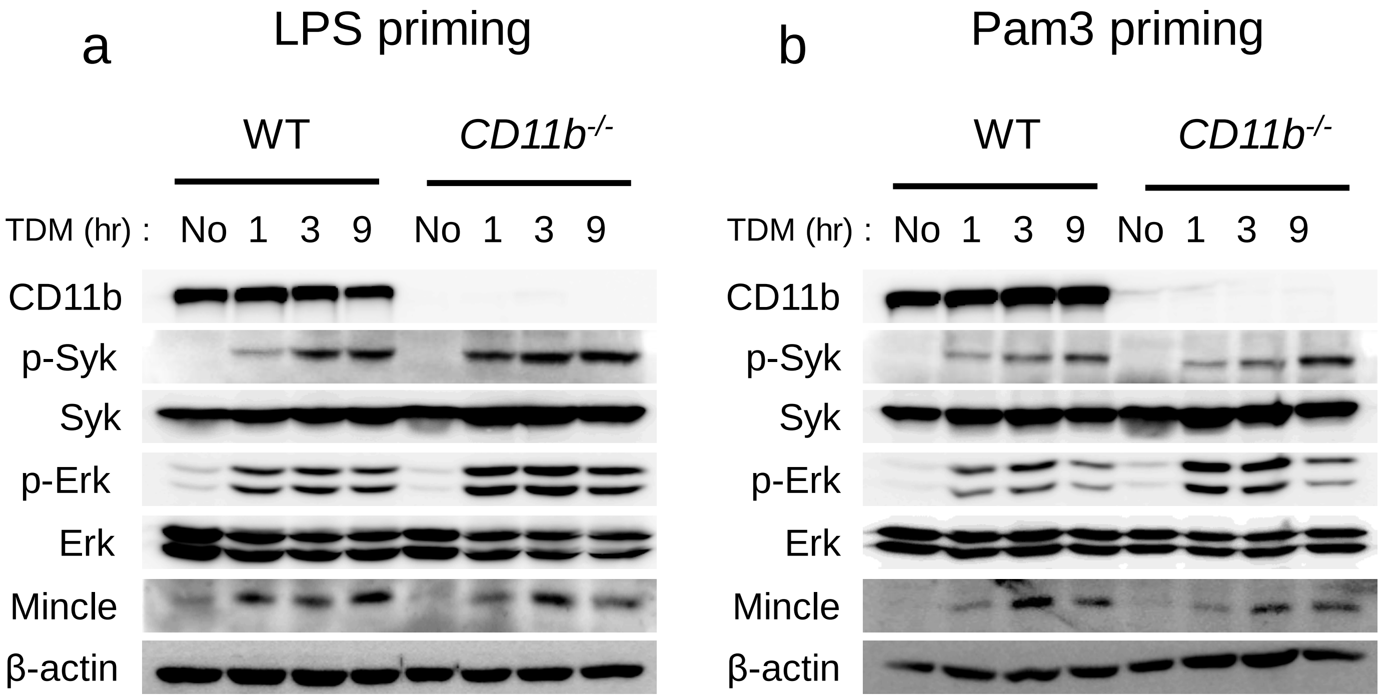

a
LPS priming
WT
CD11b-/-
TDM (hr) : No 1 3 9 No 1 3 9
CD11b
p-Syk
Syk
p-Erk
Erk
Mincle
β-actin
b
Pam3 priming
WT
CD11b-/-
TDM (hr) : No 1 3 9 No 1 3 9
CD11b
p-Syk
Syk
p-Erk
Erk
Mincle
β-actin

S Fig 4

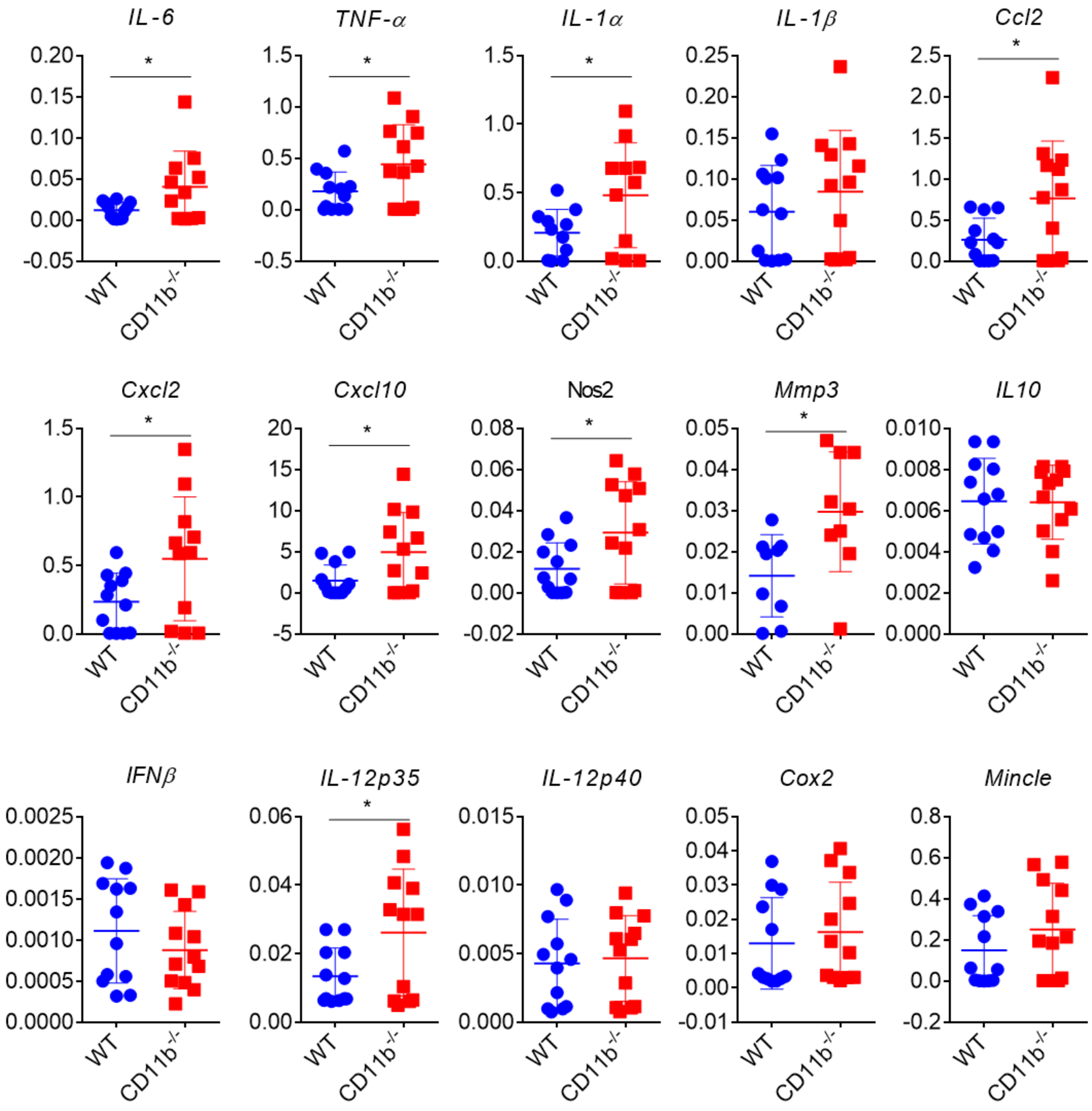

IL-6
TNF-α
IL-1α
IL-1β
Ccl2
Cxcl2
Cxcl10
Nos2
Mmp3
IL10
IFNβ
IL-12p35
IL-12p40
Cox2
Mincle
WT
CD11b-/-
*

S Fig 5

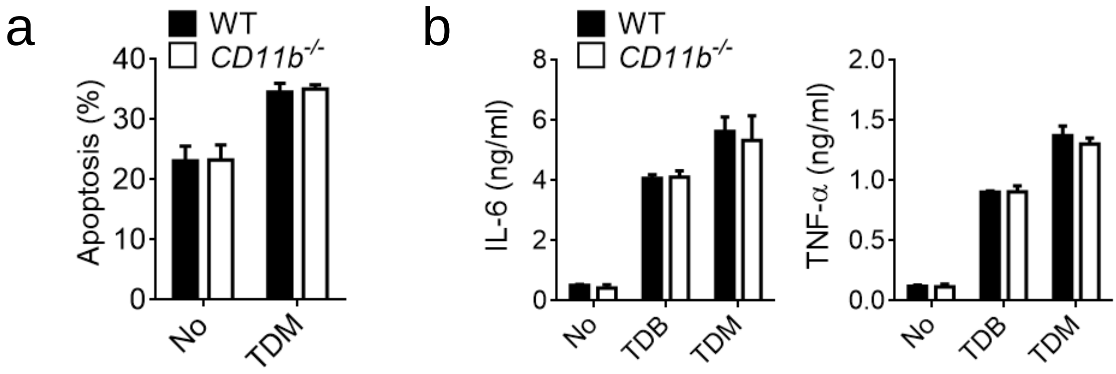
a
WT
CD11b-/-
Apoptosis (%)
0
10
20
30
40
No
TDM
b
WT
CD11b-/-
IL-6 (ng/ml)
0
2
4
6
8
No
TDB
TDM
TNF-α (ng/ml)
0.0
0.5
1.0
1.5
2.0
No
TDB
TDM

S Fig 6

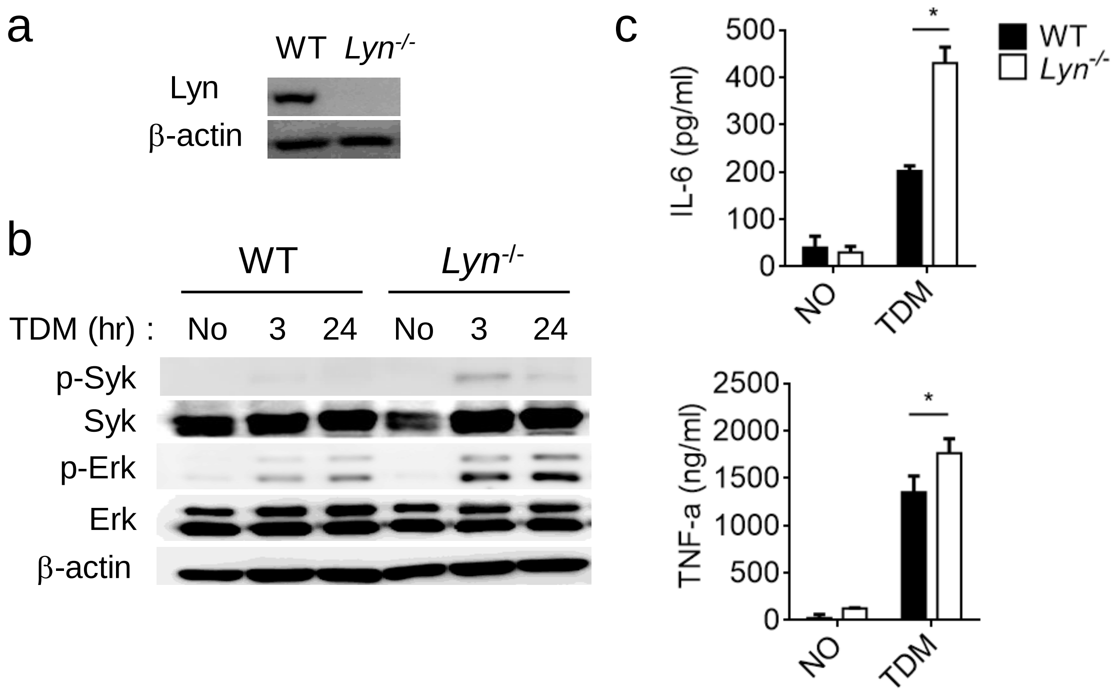

a
WT
Lyn-/-
Lyn
β-actin
b
WT
Lyn-/-
TDM (hr) :
No
3
24
No
3
24
p-Syk
Syk
p-Erk
Erk
β-actin
c
IL-6 (pg/ml)
500
400
300
200
100
0
NO
TDM
*
WT
Lyn-/-
TNF-a (ng/ml)
2500
2000
1500
1000
500
0
NO
TDM
*

S Fig 7

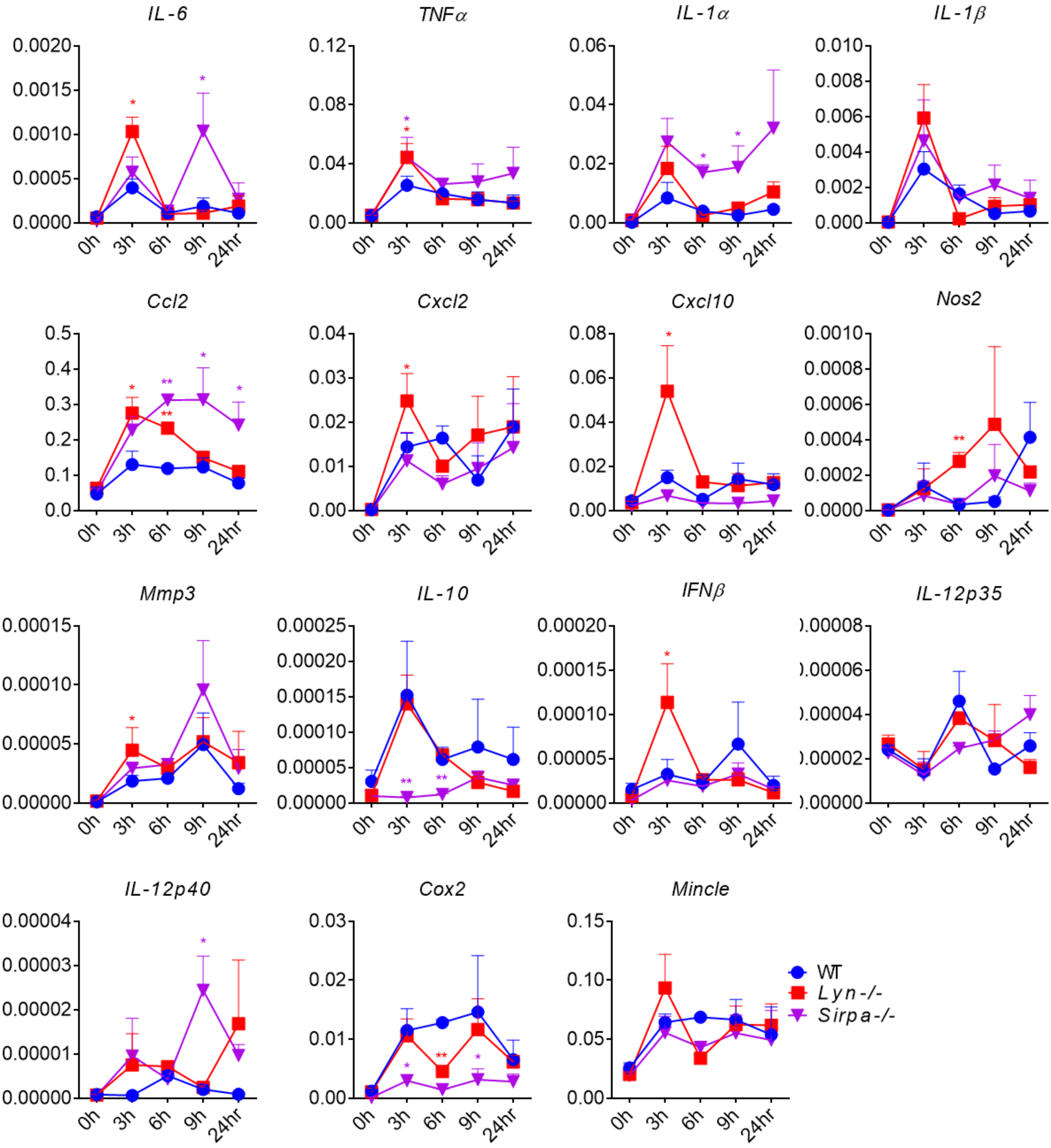

IL-6
TNFα
IL-1α
IL-1β
Ccl2
Cxcl2
Cxcl10
Nos2
Mmp3
IL-10
IFNβ
IL-12p35
IL-12p40
Cox2
Mincle
0h
3h
6h
9h
24hr
WT
Lyn-/-
Sirpa-/-

S Fig 8

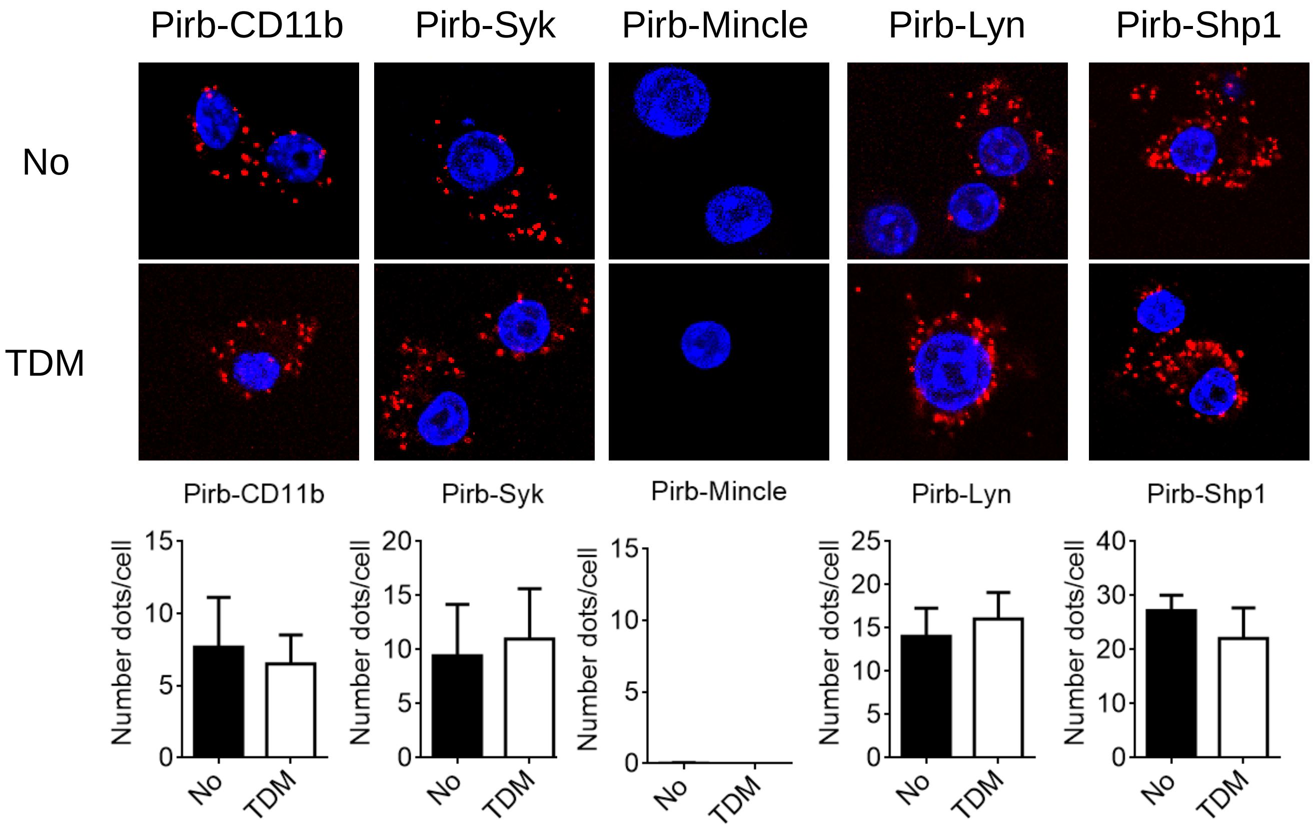
Pirb-CD11b
Pirb-Syk
Pirb-Mincle
Pirb-Lyn
Pirb-Shp1
No
TDM
Number dots/cell
No
TDM

S Fig 9

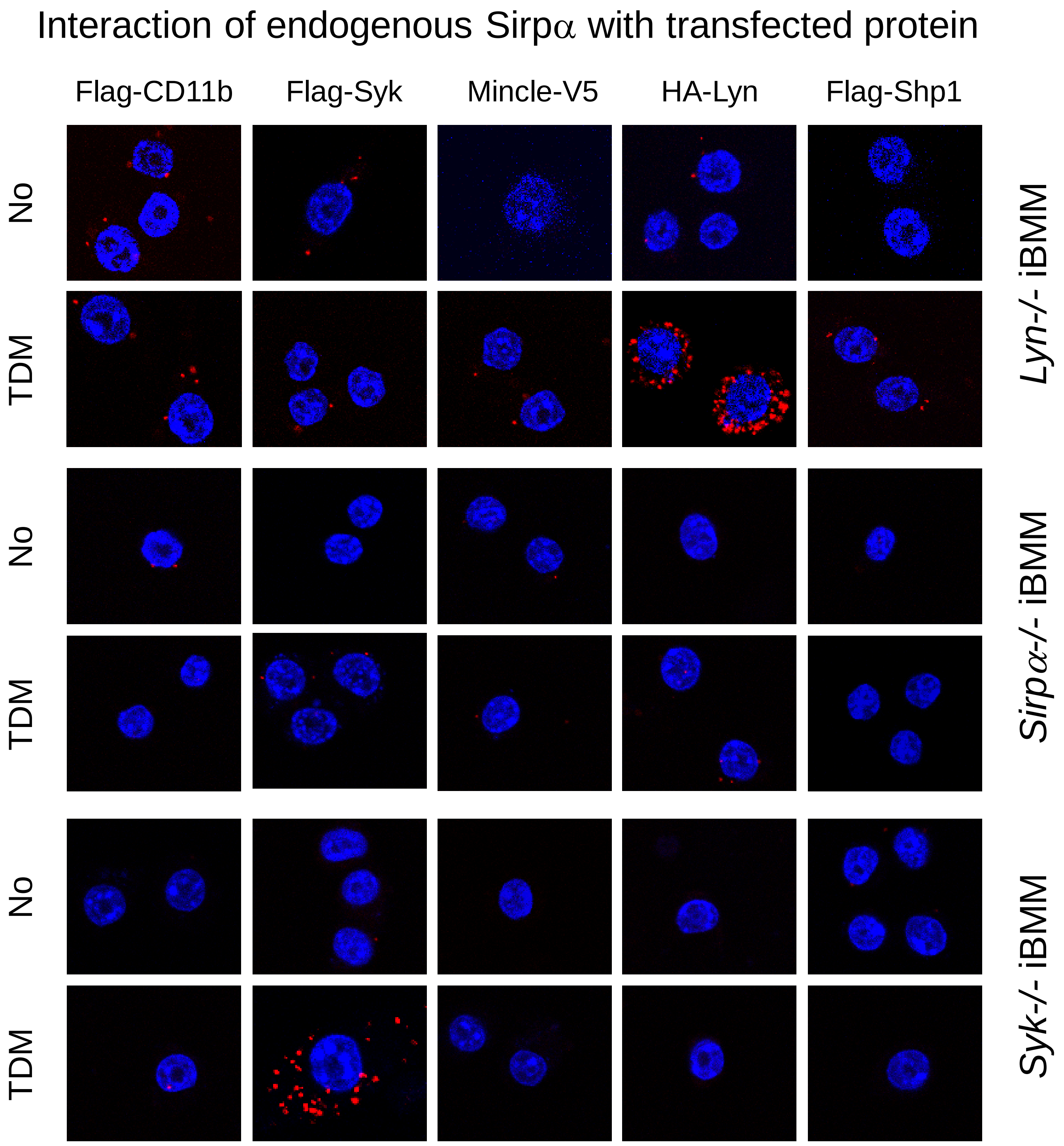
Interaction of endogenous Sirpα with transfected protein
Flag-CD11b
Flag-Syk
Mincle-V5
HA-Lyn
Flag-Shp1
No
TDM
No
TDM
No
TDM
Lyn-/- iBMM
Sirpα-/- iBMM
Syk-/- iBMM

S Fig 10

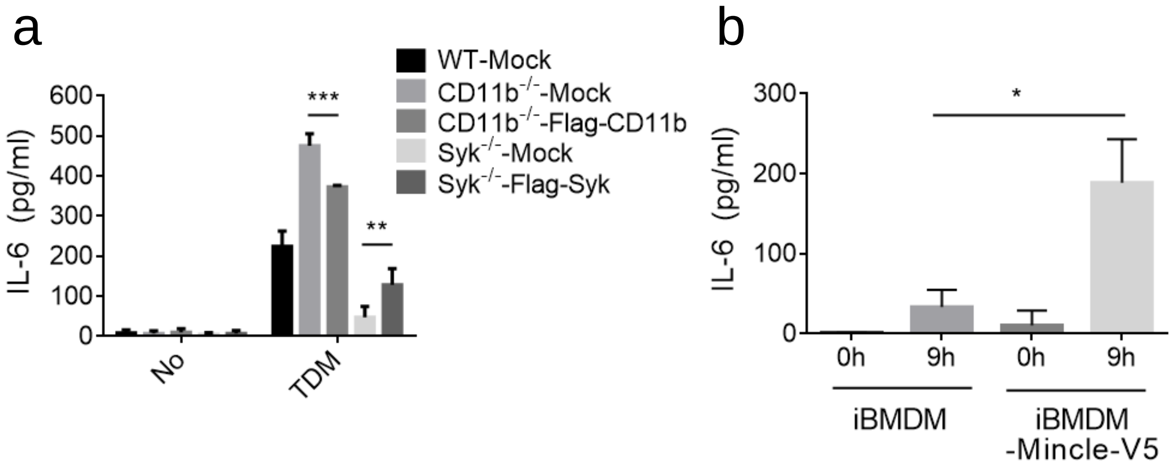
a
WT-Mock
CD11b-/--Mock
CD11b-/--Flag-CD11b
Syk-/--Mock
Syk-/--Flag-Syk
IL-6 (pg/ml)
600
500
400
300
200
100
0
***
**
No
TDM
b
IL-6 (pg/ml)
300
200
100
0
*
0h
9h
0h
9h
iBMDM
iBMDM
-Mincle-V5

S Fig 11

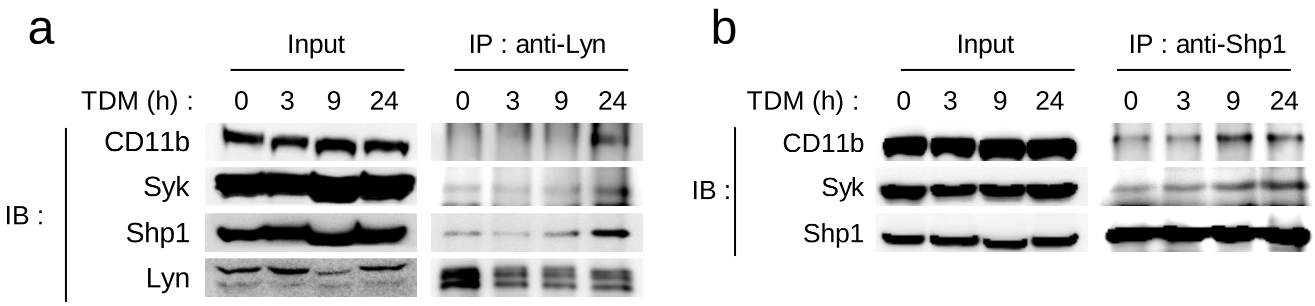
a
Input
IP : anti-Lyn
TDM (h) :
0 3 9 24
0 3 9 24
IB :
CD11b
Syk
Shp1
Lyn
b
Input
IP : anti-Shp1
TDM (h) :
0 3 9 24
0 3 9 24
IB :
CD11b
Syk
Shp1

S Fig 12

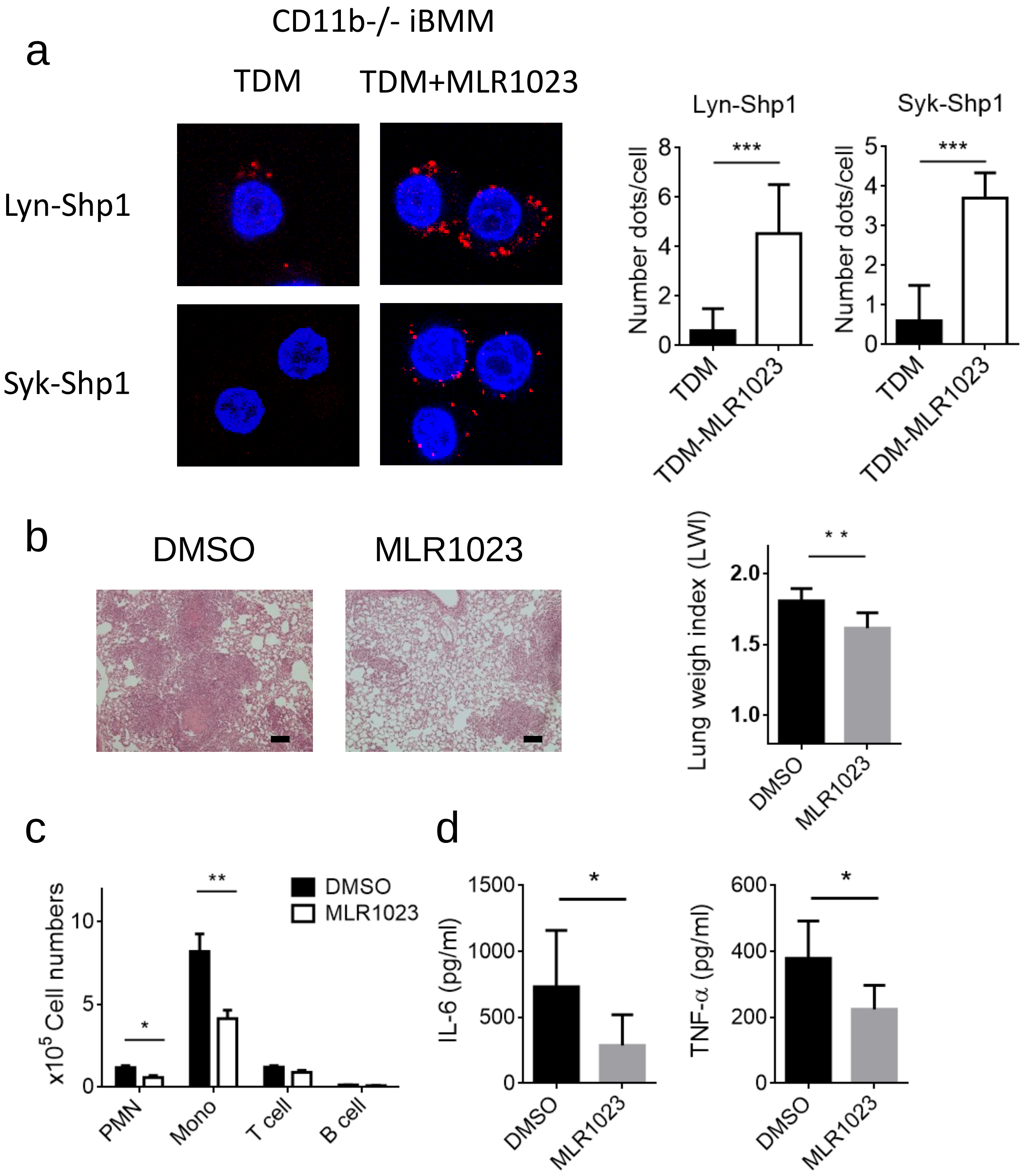

a
CD11b-/- iBMM
TDM
TDM+MLR1023
Lyn-Shp1
Syk-Shp1
Lyn-Shp1
Syk-Shp1
Number dots/cell
***
TDM
TDM-MLR1023
b
DMSO
MLR1023
Lung weigh index (LWI)
**
DMSO
MLR1023
c
x10^5 Cell numbers
DMSO
MLR1023
*
**
PMN
Mono
T cell
B cell
d
IL-6 (pg/ml)
TNF-α (pg/ml)
*
DMSO
MLR1023

S Fig 13

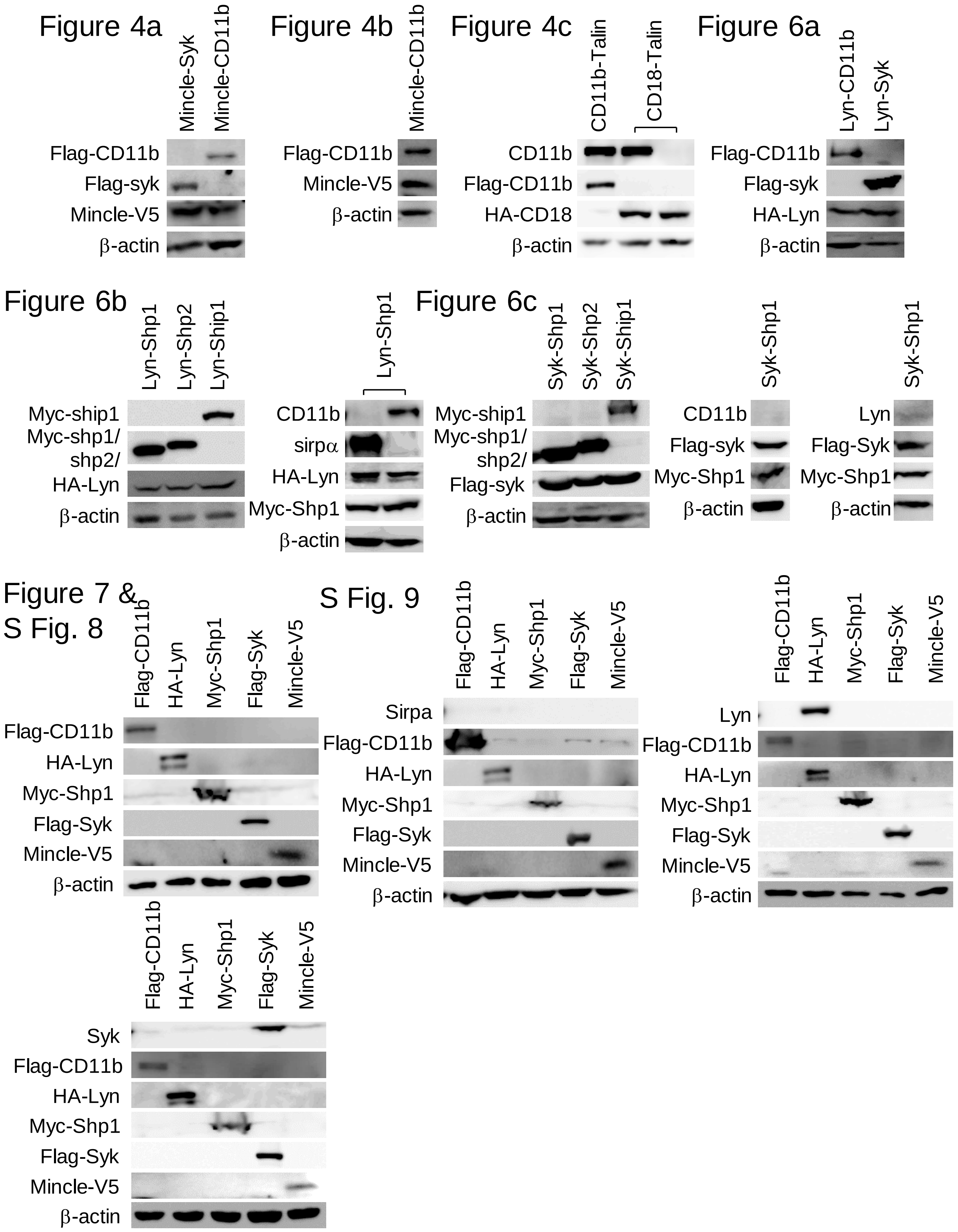
Figure 4a
Mincle-Syk
Mincle-CD11b
Flag-CD11b
Flag-syk
Mincle-V5
β-actin
Figure 4b
Mincle-CD11b
Flag-CD11b
Mincle-V5
β-actin
Figure 4c
CD11b-Talin
CD18-Talin
CD11b
Flag-CD11b
HA-CD18
β-actin
Figure 6a
Lyn-CD11b
Lyn-Syk
Flag-CD11b
Flag-syk
HA-Lyn
β-actin
Figure 6b
Lyn-Shp1
Lyn-Shp2
Lyn-Ship1
Myc-ship1
Myc-shp1/
shp2/
HA-Lyn
β-actin
Lyn-Shp1
CD11b
sirpα
HA-Lyn
Myc-Shp1
β-actin
Figure 6c
Syk-Shp1
Syk-Shp2
Syk-Ship1
Myc-ship1
Myc-shp1/
shp2/
Flag-syk
β-actin
Syk-Shp1
CD11b
Flag-syk
Myc-Shp1
β-actin
Syk-Shp1
Lyn
Flag-Syk
Myc-Shp1
β-actin
Figure 7 &
S Fig. 8
Flag-CD11b
HA-Lyn
Myc-Shp1
Flag-Syk
Mincle-V5
Flag-CD11b
HA-Lyn
Myc-Shp1
Flag-Syk
Mincle-V5
β-actin
S Fig. 9
Flag-CD11b
HA-Lyn
Myc-Shp1
Flag-Syk
Mincle-V5
Sirpa
Flag-CD11b
HA-Lyn
Myc-Shp1
Flag-Syk
Mincle-V5
β-actin
Flag-CD11b
HA-Lyn
Myc-Shp1
Flag-Syk
Mincle-V5
Lyn
Flag-CD11b
HA-Lyn
Myc-Shp1
Flag-Syk
Mincle-V5
β-actin
Flag-CD11b
HA-Lyn
Myc-Shp1
Flag-Syk
Mincle-V5
Syk
Flag-CD11b
HA-Lyn
Myc-Shp1
Flag-Syk
Mincle-V5
β-actin

Supplementary Table 1

Antibody information

Western Blot

Antibody	Clone	Distributor
anti-iNOS	#2982S	Cell Signaling
anti-Phospho-Syk (Tyr525/526)	#2710	Cell Signaling
anti-Phospho-p44/42 MAPK	#4370s	Cell Signaling
anti-Syk Antibody	#2712s	Cell Signaling
anti-Lyn (C13F9)	#2796	Cell Signaling
anti-SHPS1 (D6I3M)	#13379	Cell Signaling
anti-Erk1/2	sc-94	Cell Signaling
anti-SH-PTP1 (SHP-1)	sc-287	Santa Cruz
anti-Cox-2	sc-1745	Santa Cruz
HA-probe (Y-11)	sc-805	Santa Cruz
anti-β-Actin(N-term)	LF-PA0207	Abfrontier
anti-CD11b	ab75476	Abcam
anti-V5	ab9116	Abcam
anti-FLAG	F7425	Sigma Aldrich
anti-Myc	06-340	Millipore
anti-Mincle	D292-3	MBL life science

PLA assay

Antibody	Clone	Distributor
anti-V5 tag	ab9137	Abcam
anti-DDDDK tag	ab1257	Abcam
anti-HA tag	ab9134	Abcam
anti-Myc tag	ab9132	Abcam
anti-V5	ab9116	Abcam
anti-Talin 1	ab57758	Abcam
anti-Pirb	sc-9609	Santa Cruz
anti-SIRP-a	sc-6922	Santa Cruz
HA-probe (Y-11)	sc-805	Santa Cruz
anti-FLAG	F7425	Sigma Aldrich

Supplementary Table 2

Primers

Real time-PCR

Gene	GeneBank_ID	5'Forward sequence3'	3'Reverse sequence5'
Il-6	NM_031168	CCAAGACCATCCAATTCATC	CCACAAACTGATATGCTTAGG
TNFa	NM_013693	ATGTCCATTCCTGAGTTCTG	AATCTGGAAAGGTCTGAAGG
IL-1a	NM_010554	AGGGAGTCAACTCATTGGCG	CTTCCCGTTGCTTGACGTTG
IL-1b	NM_008361	TCAACCAACAAGTGATATTCTC	ACACAGGACAGGTATAGATTC
Cxcl2	NM_009140	CATCCCACCCACACAGTGAAAGAG	CCTTCCATGAAAGCCATCCGACTG
Ccl2	NM_011333	TTCCACAACCACCTCAAGCACTTC	TTAAGGCATCACAGTCCGAGTCAC
Cxcl10	NM_021274	TCGCTCAAGTGGCTGGGATGG	GGGCATGGCACATGGTGAAGG
Nos2	NM_010927	AATCTTGGAGCGAGTTGTGG	CAGGAAGTAGGTGAGGGCTTG
Mincle	NM_019948	AGTGAGGCATCAGGTTCAGTCAAG	GACCAGGTCAAGGTTGTCGTAGAG
Cox-2	NM_011198	GCCCGTGCTGCTCTGTCTTAAC	GTTGCTCTAGGCTTTGCTGGCTAC
Mmp3	NM_010809	CTCATGCCTATGCACCTGGA	GGCTGAGTGGTAGAGTCCCA
IL-12p35	NM_001159424	TTTGATGATGACCCTGTGCCTTGG	GATTCTGAAGTGCTGCGTTGATGG
IL-12p40	NM_001303244	AGTAAAGACATAGGTGGTATTTG	CTGGTTTCTTATCAATATCACTTC
IL-10	NM_010548	GCACTACCAAAGCCACAAAGCAG	GTCAGTAAGAGCAGGCAGCATAGC
IFNb	NM_010510	CCACTTGAAGAGCTATTACTG	AATGATGAGAAAGTTCCTGAAG
Src	NM_009271	CGCCTCACTACCGTATGTCC	CAACACCCGGAAGCCTAGAG
Yes	NM_009535	CGGCAAGACAAGGTGCAAAA	CCTGTGGAGTAACAGCCAAGT
Fyn	NM_001122893	GCCACATAGCTACAGGACCG	AATCACAGTTCGCTGGAGCA
Fgr	NM_010208	GCTGGAAGTTAGGGTTGGCT	GCCTCATAGTCGTACAGGGC
Lck	NM_001162432	ATGGAGAACGGGAGCCTAGT	GACACGCATCAATCGCAGAC
Hck	NM_001172117	CCTCCGAGATGGAAGCAAGG	GCCACTTTGGTGTGCTTGTT
Blk	NM_007549	GCCCCAGTAGAGACTCTGGA	GCCTGGGGATTTCCCATTCA
Lyn	NM_001111096	AGGAAGAACACCCTGGATGC	CATTTTCTGGGGGTTGTGCG
Frk	NM_001159544	CTATTGTGCGGCACCGAATG	AGGCAAAGGTGTCCCAAACA
Gapdh	NM_008084	GGCAAATTCAACGGCACAGTCAAG	TCGCTCCTGGAAGATGGTGATGG

Crispr-Cas9 gRNA

Gene	5'Forward sequence3'	3'Reverse sequence5'
CD11b	CACCGCTTGTGTCATGGCTTCAATC	AAACGATTGAAGCCATGACACAAGC
Lyn	CACCGGTTCGGTCAGTATTACGTAC	AAACGTACGTAATACTGACCGAACC
Sirpα	CACCGCCGCGGCCCATGGAGCCCGC	AAACGCGGGCTCCATGGGCCGCGGC
Syk	CACCGTATTGCACTACCGCATTGAC	AAACGTCAATGCGGTAGTGCAATAC

Supplementary Table 2 continued

Primers

Cloning

Plasmid		Primer sequence	Restriction Enzyme
pCS4-3×flag-shp1	Forward	AATGAATTCAATGGTGAGGTGGTTTCA	EcoRI
	Reverse	ATTCTCGAGTCACTTCCTCTTGAGAGAAC	XhoI
pCS2-6×myc-shp2	Forward	AGTGAATTCAATGACATCGCGGAGAT	EcoRI
	Reverse	TGATCTAGATCATCTGAAACTCCTCTGCT	XbaI
pCS2-6×myc-ship1	Forward	ATTGAATTCAATGCCTGCCATGGT	EcoRI
	Reverse	AAACTCGAGTCACTGCATGGCAGTC	XhoI
pCS4-3×flag-syk	Forward	AATGAATTCAATGGCGGGAAGTG	EcoRI
	Reverse	ATTCTCGAGTTAGTTAACCACGTCGTAGT	XhoI
pCS4-3×flag-CD11b	Forward	AAATCTAGATATGACTCTTAAAGCTCTTCT	XbaI
	Reverse	ATAGCTAGCTTACTGAGGTGGG	NheI
pCS4-3×flag-CD18	Forward	ATTGAATTCAATGCTGGGCCCACACT	EcoRI
	Reverse	AAACTCGAGCTAGCTTTCAGCAAACTT	XhoI
pCS4-3×HA-Lyn	Forward	GCGAGATCTGGATGGGATGTATTAAATC	BglII
	Reverse	ATTCTCGAGCTACGGTTGCTGCTGA	XhoI
pcDNA3.1-MincleV5	Forward	CCCGATCCACCATGAATTCAACCAAATCGCCTG	BamHI
	Reverse	CCGCTCGAGGTCCAGAGGACTTATTTCTGGCAT	XhoI
pcDNA4-FceR1g-Myc	Forward	CCCAAGCTTATGATCTCAGCCGTGATCTT	HindIII
	Reverse	CGCCTCGAGCTGGGGTGGTTTCTCATGCTT	XhoI

Site-directed mutagenesis

Mutation		Primer sequence
Shp1 DN C453S	Forward	GGCCCATCATTGTGCATAGCAGCGCTGGCATCGGCC
	Reverse	GGCCGATGCCAGCGCTGCTATGCACAATGATGGGCC
Shp1 DN D419A	Forward	AGTACCTGAGCTGGCCTGCCCATGGGGTTCCCAGTGAG
	Reverse	CTCACTGGGAACCCCATGGGCAGGCCAGCTCAGGTACT
