# Supplementary material for: Integrin CD11b negatively regulates Mincle-induced signaling via the Lyn–SIRPα–SHP1 complex
Source: Exp Mol Med. 2018 Feb 5;50(2):e439–. doi: 10.1038/emm.2017.256 (PMC5992981; doi:10.1038/emm.2017.256)
Supplement: Supplementary Figure Legends [file emm2017256x2.pdf]

Supplementary Figure Legends

Supplementary Figure 1. Phagocytosis of TDM-labeled beads was not altered in BMMs from WT, CD11b$^{-/-}$, or Mincle$^{-/-}$ mice.

Macrophages from WT, CD11b$^{-/-}$, and Mincle$^{-/-}$ mice were plated overnight, and GFP-labeled, uncoated or TDM-coated latex beads were added to the culture. Cells were collected at the indicated times, and internalization of the beads was analyzed by flow cytometry. Data are representative of three independent experiments.

Supplementary Figure 2. Enhanced proinflammatory gene expression in CD11b$^{-/-}$ BMMs upon TDM stimulation.

Relative mRNA expression of proinflammatory genes was quantified in WT and CD11b$^{-/-}$ BMMs treated with TDM (50 mg/ml) for the indicated times. Data are representative of three independent experiments. *$P<0.05$, **$P<0.01$, ***$P<0.0001$ (two-tailed unpaired Student's *t*-test).

Supplementary Figure 3. Mincle downstream signal activation in LPS- or Pam3-primed, TDM-simulated WT and CD11b$^{-/-}$ macrophages.

Macrophages from WT and CD11b$^{-/-}$ mice were pretreated with LPS (10 ng/ml) or Pam3 (100 ng/ml) for 3 h and then challenged with TDM for the indicated times. Levels of total and phosphorylated Syk and Erk were analyzed by immunoblot. β-actin protein expression was used as the loading control. Data are representative of two independent experiments.

Supplementary Figure 4. Induction of proinflammatory genes in the lungs of TDM-treated WT and CD11b$^{-/-}$ mice.

Expression of proinflammatory genes in lung homogenates from TDM-challenged WT and CD11b$^{-/-}$ mice lung was determined by qRT-PCR and normalized to *Gapdh*. Data are representative of two independent experiments. *P<0.05 (two-tailed unpaired Student's *t*-test).

Supplementary Figure 5. Comparison of neutrophil apoptosis and dendritic cell cytokine production upon activation of Mincle signaling in WT and CD11b$^{-/-}$ cells.

(**a**) WT and CD11b$^{-/-}$ neutrophils were treated with TDM (50 µg/ml) for 24 h. Cells were labeled with Annexin V and propidium iodide (PI) and analyzed by flow cytometry. (**b**) WT and CD11b$^{-/-}$ BMDCs were stimulated with TDM for 24 h, and the levels of TNF-α and IL-6 in the cell culture supernatants were measured by ELISA. Data are representative of three independent experiments. No significant differences were observed (P>0.05).

Supplementary Figure 6. Enhanced Mincle signaling and cytokine production in Lyn$^{-/-}$ iBMMs.

(**a**) Knockout of Lyn in iBMMs was confirmed by western blot. WT and Lyn$^{-/-}$ iBMMs were stimulated with TDM. (**b**) After 24 h of stimulation, levels of IL-6 and TNF-α were determined by ELISA. (**c**) Phosphorylation of Syk and Erk after 0, 3, or 24 h of TDM stimulation was evaluated by western blot. β-actin protein expression was used as a loading control. Data are representative of at least three (**c**) or two (**a** and **b**) independent experiments. *P<0.05 (two-tailed unpaired Student's t-test).

Supplementary Figure 7. Induction of proinflammatory genes in WT, Lyn$^{-/-}$, and SIRPα$^{-/-}$ iBMMs upon TDM stimulation.

Quantitative PCR was performed on WT, Lyn$^{-/-}$, and SIRPα$^{-/-}$ iBMMs that were treated with TDM for the indicated times. Results were normalized to *Gapdh* expression. Data are representative of three independent experiments. *P<0.05, **P<0.01 (two-tailed unpaired Student's *t*-test).

Supplementary Figure 8. Pirb does not specifically bind to CD11b, Syk, Lyn, or SHP1 following TDM stimulation.

iBMM cells were transfected separately with Flag-CD11b, Flag-Syk, V5-Mincle, HA-Lyn, or Flag-SHP1, and interactions between endogenous Pirb and the transfected targets were determined by PLA assay. Interactions were visualized as fluorescent spots (red, PLA signal). Nuclei were stained with DAPI (blue). The number of PLA signals was determined for at least 50 cells for each condition. ND, not detected. Data are representative of three independent experiments. **P<0.01, ***P<0.0001 (two-tailed unpaired Student's *t*-test).

Supplementary Figure 9. Interaction of SIRPα with transfected proteins in SIRPα$^{-/-}$, Lyn$^{-/-}$, and Syk$^{-/-}$ iBMMs.

Lyn$^{-/-}$, SIRPα$^{-/-}$, and Syk$^{-/-}$ iBMMs were transfected separately with Flag-CD11b, Flag-Syk, V5-Mincle, HA-Lyn, and Flag-SHP1, and the interactions between endogenous SIRPα and the indicated targets were determined by PLA assay. Interactions were visualized as fluorescent spots (red, PLA signal), and nuclei were stained with DAPI

(blue).

Supplementary Figure 10. IL-6 production in Mincle-expressing iBMMs, and rescue experiments with CD11b$^{-/-}$ and Syk$^{-/-}$ iBMMs.

(**a**) CD11b$^{-/-}$ iBMMs transiently transfected with Flag-CD11b and Syk$^{-/-}$ iBMMs transiently transfected with Flag-Syk were stimulated with TDM for 24 h. IL-6 secretion was determined by ELISA. (**b**) WT iBMMs and iBMMs stably expressing Mincle-V5 were treated with TDM for 9 h, and IL-6 production was assayed by ELISA. Data are representative of three independent experiments.

Supplementary Figure 11. Kinetic analysis of inhibitory complex formation upon TDM stimulation.

(**a** and **b**) Endogenous Lyn and SHP1 were immunoprecipitated from TDM-stimulated WT BMMs, and CD11b, Lyn, SHP1, and Syk in the immunoprecipitates were assayed by western blot at the indicated times. Data are representative of at least two independent experiments.

Supplementary Figure 12. MLR1023 induces the formation of inhibitory complexes in CD11b$^{-/-}$ iBMM and restricts the granuloma response in CD11b$^{-/-}$ mice treated with TDM.

(**a**) A PLA assay was conducted in CD11b$^{-/-}$ iBMM transfected for 24 h with HA-Lyn and Myc-Shp1 or Flag-Syk and Myc-SHP1, then treated with TDM (50 µg/ml) with or without MLR1023 (1 ng/ml) for another 24 h. (**b-d**) Experimental and control mice (n = 7 mice/group) were intravenously injected with TDM (2 mg/kg) in oil-in-water

emulsion on day 0. Then, mice were intraperitoneally injected with MLR1023 (6 mg/kg in PBS) or 1% DMSO in PBS every day beginning on day 1, until the mice were sacrificed 7 days post-TDM challenge. (**b**) Lung tissues were isolated and stained with hematoxylin and eosin (H&E) for histology analysis after the lung weight index (LWI) was determined. (**c**) Leukocyte subsets were analyzed by flow cytometry using distinct markers for monocytes and macrophages (Mo/Ma; CD11b+ Ly6G-), neutrophils (PMN; CD11b+ Ly6G+), T cells (CD3+), and B cells (CD19+). (**d**) The lung homogenates were analyzed by ELISA for TNF-α and IL-6. Data are representative of three (**a**) or two (**b-d**) independent experiments. **b-d**, mean and s.d. of seven mice per group. **P*<0.05, ***P*<0.01, ****P* <0.0001 (two-tailed unpaired Student's *t*-test).

Supplementary Figure 13. The expression level of transfected proteins in PLA experiments.
