# Supplementary material for: Integrin CD11b negatively regulates Mincle-induced signaling via the Lyn–SIRPα–SHP1 complex
Source: Exp Mol Med. 2018 Feb 5;50(2):e439–. doi: 10.1038/emm.2017.256 (PMC5992981; doi:10.1038/emm.2017.256)
Supplement: Supplementary Table Legends [file emm2017256x3.pdf]

Supplementary Table 1. List of primary antibodies used in western blot and PLA assay, along with the sources.

Supplementary Table 2. List of primers used in real time-PCR, CRISPR-CAS9 gRNA, cloning, and site-directed mutagenesis.

Supplementary Table 1

Antibody information

Western Blot

Antibody	Clone	Distributor
anti-iNOS	#2982S	Cell Signaling
anti-Phospho-Syk (Tyr525/526)	#2710	Cell Signaling
anti-Phospho-p44/42 MAPK	#4370s	Cell Signaling
anti-Syk Antibody	#2712s	Cell Signaling
anti-Lyn (C13F9)	#2796	Cell Signaling
anti-SHPS1 (D6I3M)	#13379	Cell Signaling
anti-Erk1/2	sc-94	Cell Signaling
anti-SH-PTP1 (SHP-1)	sc-287	Santa Cruz
anti-Cox-2	sc-1745	Santa Cruz
HA-probe (Y-11)	sc-805	Santa Cruz
anti-β-Actin(N-term)	LF-PA0207	Abfrontier
anti-CD11b	ab75476	Abcam
anti-V5	ab9116	Abcam
anti-FLAG	F7425	Sigma Aldrich
anti-Myc	06-340	Millipore
anti-Mincle	D292-3	MBL life science

PLA assay

Antibody	Clone	Distributor
anti-V5 tag	ab9137	Abcam
anti-DDDDK tag	ab1257	Abcam
anti-HA tag	ab9134	Abcam
anti-Myc tag	ab9132	Abcam
anti-V5	ab9116	Abcam
anti-Talin 1	ab57758	Abcam
anti-Pirb	sc-9609	Santa Cruz
anti-SIRP-a	sc-6922	Santa Cruz
HA-probe (Y-11)	sc-805	Santa Cruz
anti-FLAG	F7425	Sigma Aldrich

Supplementary Table 2

Primers

Real time-PCR

Gene	GeneBank_ID	5'Forward sequence3'	3'Reverse sequence5'
Il-6	NM_031168	CCAAGACCATCCAATTCATC	CCACAAACTGATATGCTTAGG
TNFa	NM_013693	ATGTCCATTCCTGAGTTCTG	AATCTGGAAAGGTCTGAAGG
IL-1a	NM_010554	AGGGAGTCAACTCATTGGCG	CTTCCCGTTGCTTGACGTTG
IL-1b	NM_008361	TCAACCAACAAGTGATATTCTC	ACACAGGACAGGTATAGATTC
Cxcl2	NM_009140	CATCCCACCCACACAGTGAAAGAG	CCTTCCATGAAAGCCATCCGACTG
Ccl2	NM_011333	TTCCACAACCACCTCAAGCACTTC	TTAAGGCATCACAGTCCGAGTCAC
Cxcl10	NM_021274	TCGCTCAAGTGGCTGGGATGG	GGGCATGGCACATGGTGAAGG
Nos2	NM_010927	AATCTTGGAGCGAGTTGTGG	CAGGAAGTAGGTGAGGGCTTG
Mincle	NM_019948	AGTGAGGCATCAGGTTCAGTCAAG	GACCAGGTCAAGGTTGTCGTAGAG
Cox-2	NM_011198	GCCCGTGCTGCTCTGTCTTAAC	GTTGCTCTAGGCTTTGCTGGCTAC
Mmp3	NM_010809	CTCATGCCTATGCACCTGGA	GGCTGAGTGGTAGAGTCCCA
IL-12p35	NM_001159424	TTTGATGATGACCCTGTGCCTTGG	GATTCTGAAGTGCTGCGTTGATGG
IL-12p40	NM_001303244	AGTAAAGACATAGGTGGTATTTG	CTGGTTTCTTATCAATATCACTTC
IL-10	NM_010548	GCACTACCAAAGCCACAAAGCAG	GTCAGTAAGAGCAGGCAGCATAGC
IFNb	NM_010510	CCACTTGAAGAGCTATTACTG	AATGATGAGAAAGTTCCTGAAG
Src	NM_009271	CGCCTCACTACCGTATGTCC	CAACACCCGGAAGCCTAGAG
Yes	NM_009535	CGGCAAGACAAGGTGCAAAA	CCTGTGGAGTAACAGCCAAGT
Fyn	NM_001122893	GCCACATAGCTACAGGACCG	AATCACAGTTCGCTGGAGCA
Fgr	NM_010208	GCTGGAAGTTAGGGTTGGCT	GCCTCATAGTCGTACAGGGC
Lck	NM_001162432	ATGGAGAACGGGAGCCTAGT	GACACGCATCAATCGCAGAC
Hck	NM_001172117	CCTCCGAGATGGAAGCAAGG	GCCACTTTGGTGTGCTTGTT
Blk	NM_007549	GCCCCAGTAGAGACTCTGGA	GCCTGGGGATTTCCCATTCA
Lyn	NM_001111096	AGGAAGAACACCCTGGATGC	CATTTTCTGGGGGTTGTGCG
Frk	NM_001159544	CTATTGTGCGGCACCGAATG	AGGCAAAGGTGTCCCAAACA
Gapdh	NM_008084	GGCAAATTCAACGGCACAGTCAAG	TCGCTCCTGGAAGATGGTGATGG

Crispr-Cas9 gRNA

Gene	5'Forward sequence3'	3'Reverse sequence5'
CD11b	CACCGCTTGTGTCATGGCTTCAATC	AAACGATTGAAGCCATGACACAAGC
Lyn	CACCGGTTCGGTCAGTATTACGTAC	AAACGTACGTAATACTGACCGAACC
Sirpα	CACCGCCGCGGCCCATGGAGCCCGC	AAACGCGGGCTCCATGGGCCGCGGC
Syk	CACCGTATTGCACTACCGCATTGAC	AAACGTCAATGCGGTAGTGCAATAC

Supplementary Table 2 continued

Primers

Cloning

Plasmid		Primer sequence	Restriction Enzyme
pCS4-3×flag-shp1	Forward	AATGAATTCAATGGTGAGGTGGTTTCA	EcoRI
	Reverse	ATTCTCGAGTCACTTCCTCTTGAGAGAAC	XhoI
pCS2-6×myc-shp2	Forward	AGTGAATTCAATGACATCGCGGAGAT	EcoRI
	Reverse	TGATCTAGATCATCTGAAACTCCTCTGCT	XbaI
pCS2-6×myc-ship1	Forward	ATTGAATTCAATGCCTGCCATGGT	EcoRI
	Reverse	AAACTCGAGTCACTGCATGGCAGTC	XhoI
pCS4-3×flag-syk	Forward	AATGAATTCAATGGCGGGAAGTG	EcoRI
	Reverse	ATTCTCGAGTTAGTTAACCACGTCGTAGT	XhoI
pCS4-3×flag-CD11b	Forward	AAATCTAGATATGACTCTTAAAGCTCTTCT	XbaI
	Reverse	ATAGCTAGCTTACTGAGGTGGG	NheI
pCS4-3×flag-CD18	Forward	ATTGAATTCAATGCTGGGCCCACACT	EcoRI
	Reverse	AAACTCGAGCTAGCTTTCAGCAAACTT	XhoI
pCS4-3×HA-Lyn	Forward	GCGAGATCTGGATGGGATGTATTAAATC	BglII
	Reverse	ATTCTCGAGCTACGGTTGCTGCTGA	XhoI
pcDNA3.1-MincleV5	Forward	CCCGATCCACCATGAATTCAACCAAATCGCCTG	BamHI
	Reverse	CCGCTCGAGGTCCAGAGGACTTATTTCTGGCAT	XhoI
pcDNA4-FceR1g-Myc	Forward	CCCAAGCTTATGATCTCAGCCGTGATCTT	HindIII
	Reverse	CGCCTCGAGCTGGGGTGGTTTCTCATGCTT	XhoI

Site-directed mutagenesis

Mutation		Primer sequence
Shp1 DN C453S	Forward	GGCCCATCATTGTGCATAGCAGCGCTGGCATCGGCC
	Reverse	GGCCGATGCCAGCGCTGCTATGCACAATGATGGGCC
Shp1 DN D419A	Forward	AGTACCTGAGCTGGCCTGCCCATGGGGTTCCCAGTGAG
	Reverse	CTCACTGGGAACCCCATGGGCAGGCCAGCTCAGGTACT
